# Effect of Physical Therapy vs Arthroscopic Partial Meniscectomy in People With Degenerative Meniscal Tears: Five-Year Follow-up of the ESCAPE Randomized Clinical Trial

**DOI:** 10.1001/jamanetworkopen.2022.20394

**Published:** 2022-07-08

**Authors:** Julia C. A. Noorduyn, Victor A. van de Graaf, Nienke W. Willigenburg, Gwendolyne G. M. Scholten-Peeters, Esther J. Kret, Rogier A. van Dijk, Rachelle Buchbinder, Gillian A. Hawker, Michel W. Coppieters, Rudolf W. Poolman

**Affiliations:** 1 Department of Orthopaedic Surgery, Joint Research, OLVG Amsterdam, Amsterdam, the Netherlands; 2 Department of Human Movement Sciences, Faculty of Behavioural and Movement Sciences, Vrije Universiteit Amsterdam, Amsterdam Movement Sciences, Amsterdam, the Netherlands; 3 Department of Orthopaedic Surgery, St Antonius Hospital Nieuwegein, the Netherlands; 4 Department of Radiology, Isala, Zwolle, the Netherlands; 5 Department of Epidemiology and Preventive Medicine, School of Public Health and Preventive Medicine, Monash-Cabrini Department of Musculoskeletal Health and Clinical Epidemiology, Cabrini Health, Melbourne, Victoria, Australia; 6 Department of Medicine, University of Toronto, Toronto, Ontario, Canada; 7 Menzies Health Institute Queensland, Griffith University, Brisbane & Gold Coast, Queensland, Australia; 8 Department of Orthopaedic Surgery, Leiden University Medical Center, Leiden, the Netherlands

## Abstract

**Importance:**

There is a paucity of high-quality evidence about the long-term effects (ie, 3-5 years and beyond) of arthroscopic partial meniscectomy vs exercise-based physical therapy for patients with degenerative meniscal tears.

**Objectives:**

To compare the 5-year effectiveness of arthroscopic partial meniscectomy and exercise-based physical therapy on patient-reported knee function and progression of knee osteoarthritis in patients with a degenerative meniscal tear.

**Design, Setting, and Participants:**

A noninferiority, multicenter randomized clinical trial was conducted in the orthopedic departments of 9 hospitals in the Netherlands. A total of 321 patients aged 45 to 70 years with a degenerative meniscal tear participated. Data collection took place between July 12, 2013, and December 4, 2020.

**Interventions:**

Patients were randomly allocated to arthroscopic partial meniscectomy or 16 sessions of exercise-based physical therapy.

**Main Outcomes and Measures:**

The primary outcome was patient-reported knee function (International Knee Documentation Committee Subjective Knee Form (range, 0 [worst] to 100 [best]) during 5 years of follow-up based on the intention-to-treat principle, with a noninferiority threshold of 11 points. The secondary outcome was progression in knee osteoarthritis shown on radiographic images in both treatment groups.

**Results:**

Of 321 patients (mean [SD] age, 58 [6.6] years; 161 women [50.2%]), 278 patients (87.1%) completed the 5-year follow-up with a mean follow-up time of 61.8 months (range, 58.8-69.5 months). From baseline to 5-year follow-up, the mean (SD) improvement was 29.6 (18.7) points in the surgery group and 25.1 (17.8) points in the physical therapy group. The crude between-group difference was 3.5 points (95% CI, 0.7-6.3 points; *P* < .001 for noninferiority). The 95% CI did not exceed the noninferiority threshold of 11 points. Comparable rates of progression of radiographic-demonstrated knee osteoarthritis were noted between both treatments.

**Conclusions and Relevance:**

In this noninferiority randomized clinical trial after 5 years, exercise-based physical therapy remained noninferior to arthroscopic partial meniscectomy for patient-reported knee function. Physical therapy should therefore be the preferred treatment over surgery for degenerative meniscal tears. These results can assist in the development and updating of current guideline recommendations about treatment for patients with a degenerative meniscal tear.

**Trial Registration:**

ClinicalTrials.gov Identifier: NCT01850719

## Key Points

**Question:**

Is exercise-based physical therapy noninferior to arthroscopic partial meniscectomy during a 5-year follow-up period in patients aged 45 to 70 years with a degenerative meniscal tear?

**Findings:**

In this noninferiority randomized clinical trial, no significant or clinically relevant between-group difference in patient-reported knee function was noted on the International Knee Documentation Committee Subjective Knee Form at the 5-year follow-up. Physical therapy was not inferior to arthroscopic partial meniscectomy.

**Meaning:**

The findings of this trial support the recommendation that exercise-based physical therapy should be the preferred treatment over surgery for degenerative meniscal tears.

## Teaser

The randomized clinical trial compares the use of exercise-based physical therapy with surgery in patients with a degenerative meniscal tear.

## Introduction

Randomized clinical trials (RCTs) and their aggregated data in systematic reviews show that arthroscopic partial meniscectomy has no clinically meaningful patient benefit compared with exercise therapy in patients with a degenerative meniscal tear in the first 2 years of follow-up.^1,2,3,4,5,6,7,8^ These findings have been embedded in the most recently updated guidelines.^9,10,11^

Long-term trial results (ie, 3- to 5-year follow-up) of arthroscopic partial meniscectomy vs exercise therapy for patients with degenerative meniscal tears have been published.^12,13,14,15,16^ These studies have consistently reported a lack of clinically relevant differences between partial meniscectomy and exercise therapy on important patient-reported outcomes, such as knee function. While these results are consistent, debate still exists on the progression of osteoarthritis (OA) after arthroscopic partial meniscectomy.^17,18,19^ The long-term trial results have reported conflicting data with respect to this outcome.^12,13,14,15,16^ The RCT conducted by Sihvonen et al^15^ found that arthroscopic partial meniscectomy is associated with a slightly increased risk of radiographic knee OA compared with exercise therapy. The study by Katz et al^14^ found a 5 times higher risk for total knee replacement (ie, the treatment for end-stage knee OA) after surgery vs exercise-based physical therapy. However, the trials by Berg et al,^12^ Herrlin et al,^13^ and Sonesson et al^16^ that compared surgery with exercise therapy found no clinically relevant difference between the 2 treatments for OA progression.

Although the current evidence suggests nonoperative management is best in patients with degenerative meniscal tears, it has not yet led to a substantial reduction of meniscal surgeries for this population.^20^ Additional evidence from RCTs on the long-term outcomes (ie, ≥3 years) of patients with degenerative meniscal tears is likely to further clarify the role of surgery and exercise in the management of meniscal tears.

The primary aim of this study was to compare patient-reported knee function at the 5-year follow-up after arthroscopic partial meniscectomy and exercise-based physical therapy in patients with a degenerative meniscal tear. The secondary aim was to assess the progression of radiographic and symptomatic evidence of knee OA. We hypothesized that exercise-based physical therapy is noninferior to arthroscopic partial meniscectomy over a period of 5 years.

## Methods

### Design

We performed a 5-year follow-up assessment of patients in the ESCAPE trial, a multicenter RCT comparing arthroscopic partial meniscectomy with exercise-based physical therapy.^6,21^ The primary end point of the ESCAPE trial was at 2 years. The published protocol and 2-year results^6,21^ contain a detailed description of the design and methods of the trial, including the sample size calculation, and the study protocol is presented in Supplement 1. We added our 5-year follow-up statistical analysis plan to the trial registration on October 6, 2021, before data analyses commenced.

The Medical Ethical Committee–United approved the ESCAPE trial in 2013, including the data collection for the 5-year follow-up. All patients provided written informed consent prior to participating in the trial. We followed the Consolidated Standards of Reporting Trials (CONSORT) reporting guideline.

### Participants

We recruited patients from 9 participating orthopedic departments of secondary and tertiary care hospitals in the Netherlands. Patients had to be between ages 45 and 70 years and have a symptomatic, degenerative, magnetic resonance imaging–confirmed meniscal tear. We excluded patients with a locked knee or trauma requiring acute surgery, associated injuries on the index knee (symptomatic partial or total anterior or posterior cruciate ligament rupture), severe structural knee OA (grade 4 on the Kellgren-Lawrence [KL] Grading Scale), or a body mass index greater than 35 (calculated as weight in kilograms divided by height in meters squared). Patients did not receive financial compensation for participating in the study. The 16 physical therapy sessions were compensated for patients allocated to physical therapy because this therapy is not reimbursed by the basic Dutch health insurance. A more detailed description of the selection criteria is presented in the protocol.^21^

### Randomization and Blinding

We enrolled all patients between July 12, 2013, and November 5, 2015. The 5-year follow-up evaluation was completed December 4, 2020. We randomized patients to arthroscopic partial meniscectomy or exercise-based physical therapy using a computerized randomization schedule with a 1:1 ratio and varying block sizes up to a maximum of 6. The randomization scheme was stratified by hospital and age (45-57 and 58-70 years). Owing to practical considerations, patients, clinicians, and research staff, with the exception of the radiologist in charge of examining the radiographic images, were not blinded to treatment allocation during data collection. We, however, performed the analyses and interpreted the results based on data that were blinded for treatment allocation. We unblinded the treatment allocation after we reached consensus on interpretation of the results.

### Intervention

#### Arthroscopic Partial Meniscectomy

Patients allocated to arthroscopic partial meniscectomy received arthroscopic partial meniscectomy within 4 weeks after randomization at the hospital of inclusion. The arthroscopic partial meniscectomy included a standardized intra-articular inspection of the knee joint, including assessment of the lateral and medial meniscus, the anterior cruciate ligament, the level of chondropathy, and a general classification of the level of cartilage degeneration. The surgeon removed the affected part of the meniscus until a stable and solid meniscus remained. The costs for surgery were covered by the patients’ health insurance. After surgery, all patients received written postoperative instructions, including a home exercise program. Eight weeks after surgery, patients visited the outpatient orthopedic clinic for a clinical consultation. According to the guidelines of the Dutch Orthopedic Association, we referred patients for physical therapy only in case of delayed recovery.^10^

#### Exercise-Based Physical Therapy

Patients allocated to physical therapy were referred to participating physical therapy practices and started exercise therapy within 2 weeks of randomization. The treatment protocol consisted of a physical therapist–led incremental exercise program over a period of 8 weeks, consisting of 16 sessions of 30 minutes each. If knee symptoms persisted following the physical therapy program (eg, knee pain, limitations in daily activities, or mechanical dysfunction), the patient could attend additional physical therapy sessions or opt for arthroscopic partial meniscectomy based on a shared decision after consultation with the orthopedic surgeon. A detailed description of the physical therapy protocol can be found in the eAppendix in Supplement 2.

### Data Collection

Patients completed self-administered questionnaires at baseline and 3 months, 6 months, 1 year, 2 years, and 5 years after enrollment. Weight-bearing radiographic images were performed at baseline and 5-year follow-up.

Patients completed the questionnaires either online or on paper according to their preference. Baseline data included patient characteristics, the level of OA assessed on radiographic images, and several patient-reported outcome measures. Each item in the online questionnaires required an answer to limit missing data. For the paper-based questionnaires, the researcher tried to retrieve missing items by telephone. To optimize the response rate, patients received up to 3 reminders. If a patient was not able or willing to complete a questionnaire at a specific time point, efforts were made to collect data for the subsequent time points.

### Primary Outcome Measure

The primary outcome was the difference between the surgery group and physical therapy group in patient-reported knee function, quantified by the International Knee Documentation Committee (IKDC) Subjective Knee Form questionnaire over a period of 5 years. The IKDC questionnaire assesses knee-specific symptoms, function, and sports activity and was developed for patients with knee ligament or meniscal injuries.^22^ In patients with a meniscal tear, the IKDC questionnaire is a reliable, valid, and responsive measurement instrument to assess knee function.^23,24^ The score ranges from 0, representing the worst knee function, to 100, indicating no limitations in functioning. The minimal important change for people with degenerative meniscal tears is 11 points.^23^

### Secondary Outcome Measures

Secondary outcomes included the progression of knee OA assessed on radiographic images and additional patient-reported outcomes. All radiographic images were taken with the patient in a standing position and with an anterior-posterior view. An experienced radiologist (R.A.v.d.D.) blinded to treatment allocation performed all radiographic evaluations to assess the presence and grade of knee OA using the KL scale, ranging from 0 (no knee OA) to 4 (severe knee OA),^25^ as well as the Osteoarthritis Research Society International (OARSI) atlas sum score,^26^ a semiquantitative instrument that assesses the severity of joint space narrowing and osteophytes in knee OA.

We specifically chose to distinguish between radiographic knee OA, looking only at the structural changes of cartilage tissue, and symptomatic knee OA, combining structural changes on radiographic images with the patient-reported symptoms.

#### Radiographic Knee OA

Using the OARSI atlas sum score, we assessed the severity of knee osteophytes at baseline and at the 5-year follow-up for the medial and lateral femoral condyle and medial and lateral tibia plateau. Joint space narrowing was assessed for the medial and lateral compartments. The severity for each item was scored with an ordered categorical grade (grade 0, normal; grade 1, mild change in joint space or osteophytes; grade 2, moderate change in joint space or osteophytes; and grade 3, severe change in joint space or osteophytes). We calculated a sum score by adding the scores of all items. We defined radiographic knee OA if at least 1 of the following criteria was met: (1) joint space narrowing grade 2 or higher, (2) sum of osteophyte grades greater than or equal to 2, or (3) grade 1 joint space narrowing in combination with 1 or more grade 1 osteophytes.^12^

To determine the progression of knee OA between baseline and 5-year follow-up, we used the OARSI sum score (range, 0-18) of the 6 items. Patients who underwent partial or total knee replacement surgery received the score of end-stage knee OA (OARSI score of 3 for the involved components).

#### Symptomatic Knee OA

We planned to assess symptomatic knee OA at the 5-year follow-up but found no consensus on cutoff values for symptomatic knee OA in the literature. We therefore introduced a pragmatic definition based on radiographic images and the Patient Acceptable Symptom State of the Knee Osteoarthritis Outcome Score-Physical Functioning Short-Form (KOOS-PS) score (range, 0 [best] to 100 [worst] physical functioning). The KOOS-PS is a reliable, valid, and responsive measurement instrument to assess physical functioning in patients with knee OA.^27,28^ Symptomatic knee OA was considered to be present in patients with both a KL score greater than or equal to 2^25^ and KOOS-PS score exceeding the Patient Acceptable Symptom State of 52.8 points for people with knee OA.^29^ The data manager (E.J.K.) combined the KL score, assessed by the radiologist, and the patient-reported KOOS-PS score into a symptomatic knee OA (yes or no) score using syntax coded in SPSS (IBM SPSS).

#### Additional Patient-Reported Outcomes

Additional patient-reported outcomes were (1) pain intensity during activities, assessed for the preceding week and scored using a visual analog scale ranging from 0 (no pain) to 100 (worst imaginable pain); (2) physical function using the KOOS-PS; and (3) quality of life, assessed with the EuroQol 5 Dimension 5 Level, which is a widely used instrument for health-related quality-of-life based on 5 dimensions: mobility, self-care, daily activities, pain/discomfort, and depression/anxiety.^30^ These 5 dimensions were combined into a health state. The index score ranges from 0 (death) to 1 (best quality of life). We assessed pain intensity and quality of life at baseline, 3 months, 6 months, 1 year, 2 years, and 5 years and physical function using the KOOS-PS only at the 5-year follow-up.

#### Adverse Events and Additional Knee Surgery

The adverse events up to the 2-year follow-up were previously reported.^6^ In the 5-year follow-up questionnaire, we asked patients: “Did you have additional knee surgery performed on your affected knee in the last 3 years?” If yes, patients were asked to specify the type of surgery (arthroscopic partial meniscectomy, total knee replacement, partial knee replacement, cartilage surgery, or other). We reported these additional knee surgeries descriptively.

### Sample Size Calculation

We calculated the study sample size before the trial in 2013. The sample size was calculated for the primary end point, which was the 2-year follow-up. We based our sample size on an SD of 18 points on the IKDC questionnaire, a power of 90%, a 2-sided α of .05, and a noninferiority margin of 8 points on the IKDC questionnaire. With an anticipated 20% loss to follow-up and a 25% delayed arthroscopic partial meniscectomy rate after 24 months, 160 participants per treatment group were needed.

### Statistical Analysis

We used descriptive statistics to report baseline characteristics of the study population and frequencies of further surgeries, partial or total knee arthroplasties, and patients who received delayed surgery following physical therapy. Similar to our previous analyses^6^ and as recommended for clinical trials, we analyzed 5-year follow-up data using the intention-to-treat principle. In the intention-to-treat analysis, patients were analyzed in 2 groups according to their randomly allocated treatment. To test for robustness of the results regarding knee function and radiographic knee OA, we also performed an as-treated analysis. For this process, we divided patients into 3 groups: (1) patients allocated to arthroscopic partial meniscectomy who underwent surgery, (2) patients allocated to physical therapy who completed 16 or more physical therapy sessions, and (3) patients allocated to physical therapy who underwent arthroscopic partial meniscectomy during the 5-year follow-up. Patients who did not undergo their allocated treatment (either surgery or completion of physical therapy) were excluded from the as-treated analysis.

We analyzed a continuous outcome measure using linear mixed-model analyses with a random intercept. We defined the overall crude intervention effects by a model with only treatment group and the baseline value of the outcome as independent variables. We added time and time-by-treatment interaction terms to specify crude intervention effects for each follow-up time point. Adjusted intervention effects were calculated using similar models expanded with the following potential confounders as independent variables^6^: level of OA at baseline using the KL classification,^25^ baseline pain during weight bearing, body mass index at baseline (<25, 25-30, or >30-35), and sex. In all models, physical therapy was defined as the reference treatment. We tested for noninferiority based on a 1-sample z test with respect to the noninferiority threshold of 11 points and 1-sided α level of 0.025. Statistical significance was assessed at the .05 level for secondary outcome measures. Significant *P* values indicate noninferiority, ie, the upper limit of the 95% CI of the between-group difference does not exceed the noninferiority threshold of 11 points. We report the secondary outcomes for knee OA descriptively. All analyses were performed using SPSS, version 27 (IBM SPSS).

## Results

### Patients

We included and randomized a total of 321 patients (mean [SD] age, 58 [6.6] years; 161 women [50.2%], 160 men [49.8%]) to either surgery (n = 159) or physical therapy (n = 162). Directly after randomization, 1 patient in each group withdrew from participation. After 5 years, 278 participants (87.1%) completed the follow-up: 139 in each group, with a mean follow-up time of 61.8 months (range, 58.8-69.5 months). In the surgery arm, a total of 19 patients (12.0%) did not complete the follow-up at 5 years. In the physical therapy arm, a total of 22 patients (13.6%) did not complete the follow-up at 5 years. In 4 patients (1 physical therapy vs 3 surgery), the loss to follow-up was related to the lack of knee symptoms. Other reasons for loss to follow-up were patients did not respond to the questionnaires and reminders (8 physical therapy vs 6 surgery), patients did not wish to complete the follow-up (0 physical therapy vs 2 surgery), the reason was unrelated to knee symptoms (1 physical therapy vs 3 surgery), or the reason was unknown (12 physical therapy vs 5 surgery).

**Figure 1.**
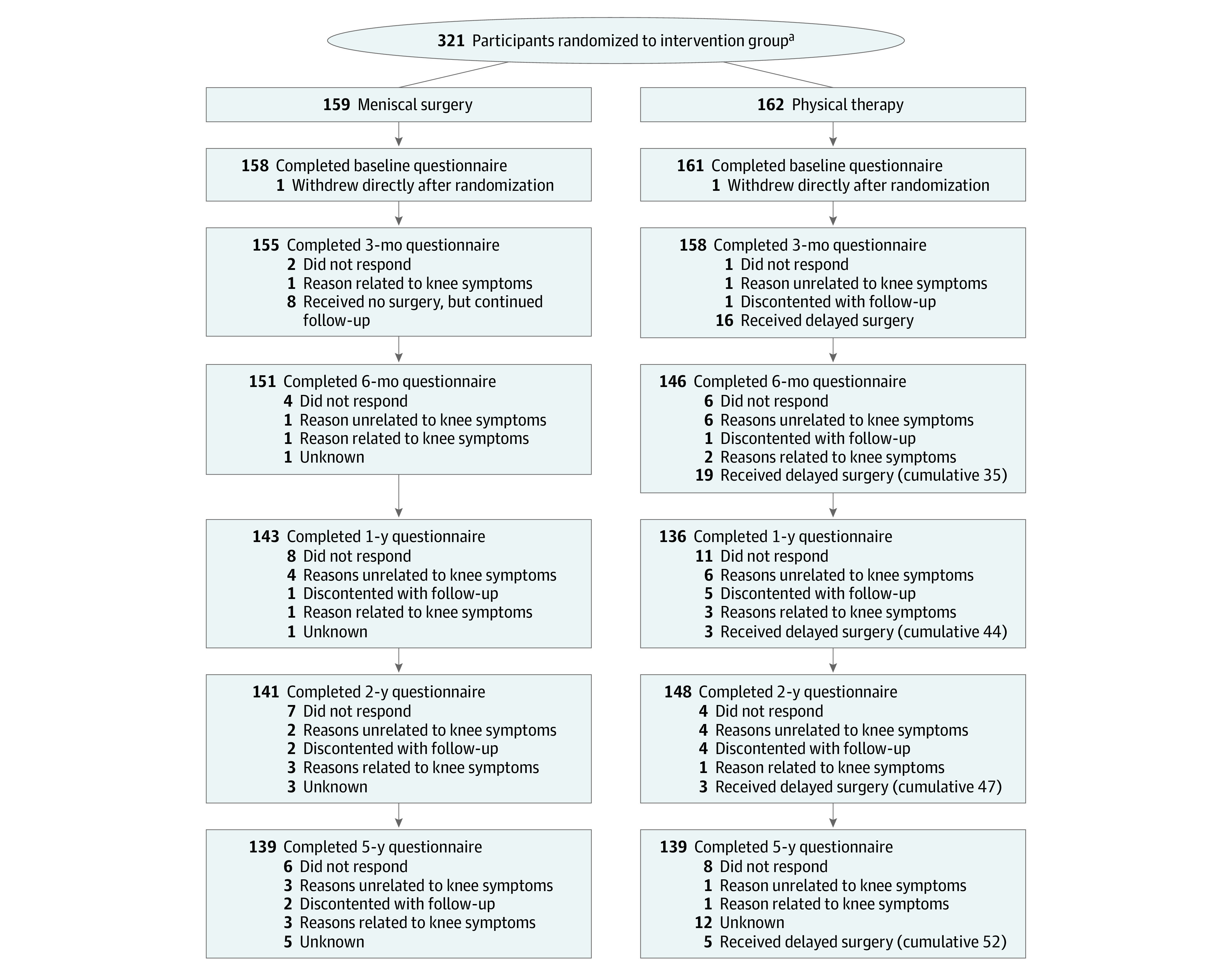
Flow of Patients ^a^The number of patients screened for eligibility was not available. The flow diagram represents separate time points instead of a mathematical flow.

Figure 1 presents the patient flow through the trial, and Table 1 reports the baseline characteristics for the surgery and physical therapy groups. The groups had similar baseline characteristics. During the follow-up period, 52 of 162 participants (32.1%) in the physical therapy group underwent delayed arthroscopic partial meniscectomy due to persistence of knee symptoms: 44 patients within the first 2 years of follow-up and 5 patients within the last 3 years of the trial (Figure 1).

**Table 1.** Baseline Characteristics

Characteristic	No. (%)	
	Arthroscopic partial meniscectomy group (n = 158)	Physical therapy group (n = 161)
Demographic		
Age, mean (SD), y	57.6 (6.5)	57.3 (6.8)
Sex		
Men	78 (49.4)	80 (49.7)
Women	80 (50.6)	81 (50.3)
Treated knee, right side	88 (55.7)	81 (50.3)
Educational level, high^a^	67 (42.4)	86 (53.4)
BMI, mean (SD)	26.7 (3.8)	27.2 (4.0)
18.5-25	56 (35.4)	53 (32.9)
25-30	72 (45.6)	67 (41.6)
30-35	30 (19.0)	41 (25.5)
Mechanical problems^b^	56 (35.4)	67 (41.6)
Imaging		
Affected meniscus on MRI		
Medial	126 (79.7)	136 (84.5)
Lateral	30 (19.0)	25 (15.5)
Both	2 (1.3)	0
OA score on radiographic images, No.	148	146
OARSI sum score, mean (SD)	1.9 (1.5)	2.1 (1.6)
Kellgren-Lawrence classification^c^		
0 (no OA)	18 (12.0)	15 (10.1)
1 (doubtful)	81 (54.0)	74 (49.7)
2 (minimal OA)	45 (30.0)	55 (36.9)
3 (moderate OA)	6 (4.0)	5 (3.3)
4 (severe OA)^d^	0	0
Patient-reported outcomes, mean (SD)		
IKDC score	44.8 (16.6)	46.5 (14.6)
Pain during activities	61.1 (24.5)	59.3 (22.6)

Abbreviations: BMI, body mass index (calculated as weight in kilograms divided by height in meters squared); IKDC, International Knee Documentation Committee; MRI, magnetic resonance imaging; OA, osteoarthritis; OARSI, Osteoarthritis Research Society International.

^a^ Educational level was measured according to the International Standard Classification of Education (ISCED) and dichotomized to low (ISCED level 0-3; eg, early childhood education, primary education, or high school) or high (ISCED level 4-8; eg, any education beyond high school, including bachelor’s, master’s, or doctoral degree).

^b^ In contrast to locking of the knee joint, which was an exclusion criterion, mechanical problems, such as catching and clicking of the knee, were allowed for inclusion.

^c^ Osteoarthritis was assessed using standing radiographic images of the knee in the anterior-posterior direction.

^d^ Patients with a Kellgren-Lawrence grade of 4 on baseline radiographic images were excluded from the trial.

In the as-treated analyses, we excluded a total of 25 participants (7.8%): 8 allocated to surgery withdrew from surgery and 17 allocated to physical therapy did not adhere to the treatment protocol. The as-treated analysis therefore included the data of 150 participants in the surgery group, 92 participants in the physical therapy, and 52 participants in the delayed surgery group.

### Primary Outcome Measure—Patient Reported Knee Function

#### Intention-to-Treat Analysis

**Figure 2.**
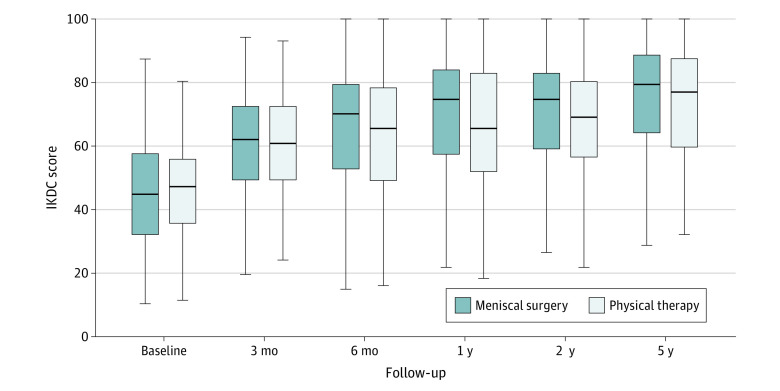
International Knee Documentation Committee (IKDC) Subjective Knee Form Questionnaire Scores During Follow-up The IKDC score for knee function per time point, shown with the box representing the IQR and median score indicated as the line within the box. The error bars indicate the 95% CIs.

The crude and adjusted between-group differences in effect between physical therapy and arthroscopic partial meniscectomy for knee function overall and at each time point are reported in Table 2, and the knee function box plots in each group at each time point are displayed in Figure 2. Over the 5 years of follow-up, the overall crude between-group difference was 3.5 points (95% CI, 0.7-6.3 points; *P* < .001 for noninferiority) and was 3.8 points (95% CI, 0.8-6.8; *P* < .001 for noninferiority) after adjusting for confounding factors. From baseline to the 5-year follow-up, the surgery group had a mean (SD) improvement of 29.6 (18.7) points (from 44.8 [16.6] to 74.7 [18.4] points), and the exercise-based physical therapy group had a mean improvement of 25.1 (17.8) points (from 46.5 [14.6] to 73.1 [17.7] points) on the IKDC questionnaire score for knee function.

**Table 2.** Crude and Adjusted Between-Group Differences in Effect for Knee Function Overall and at Each Time Point

Variable	Intention-to-treat and surgery vs physical therapy		As-treated analyses^a^			
	Between-group difference (95% CI)^b^	*P* value for noninferiority^c^	Surgery vs physical therapy		Delayed surgery vs physical therapy	
			Between-group difference (95% CI)	*P* value for noninferiority^c^	Between-group difference (95% CI)	*P* value for noninferiority^c^
**Crude difference**						
3 mo	0.8 (−2.8 to 4.3)	<.001	−2.4 (−6.4 to 1.7)	<.001	−9.1 (−14.6 to −3.6)	<.001
6 mo	3.4 (−0.2 to 7.1)	<.001	2.1 (−2.0 to 6.2)	<.001	−5.4 (−11.2 to 0.3)	<.001
1 y	5.7 (2.0 to 9.5)	.003	5.7 (1.6 to 9.9)	.007	−0.6 (−6.5 to 5.4)	<.001
2 y	5.0 (1.4 to 8.7)	.001	4.1 (−0.1 to 8.3)	.001	−2.5 (−8.3 to 3.2)	<.001
5 y	2.8 (−0.9 to 6.5)	<.001	3.0 (−1.2 to 7.1)	<.001	2.3 (−3.7 to 8.2)	.002
Overall^d^	3.5 (0.7 to 6.3)	<.001	2.4 (−0.8 to 5.5)	<.001	−3.8 (−8.2 to 0.6)	<.001
**Adjusted difference**						
3 mo	1.0 (−2.9 to 4.8)	<.001	−2.3 (−6.7 to 2.0)	<.001	−9.7 (−15.7 to −3.7)	<.001
6 mo	4.1 (0.1 to 8.0)	<.001	2.6 (−1.8 to 7.1)	<.001	−6.1 (−12.4 to 0.2)	<.001
1 y	7.1 (3.0 to 11.1)	.03	6.6 (2.1 to 11.1)	.03	−2.4 (−8.9 to 4.2)	<.001
2 y	5.5 (1.5 to 9.5)	.003	4.0 (−0.5 to 8.5)	.001	−4.6 (−10.9 to −1.7)	<.001
5 y	3.4 (−0.7 to 7.4)	<.001	3.1 (−1.4 to 7.6)	<.001	0.9 (−5.5 to 7.4)	.001
Overall^d^	3.8 (0.8 to 6.8)	<.001	2.2 (−1.2 to 5.6)	<.001	−4.9 (−9.6 to −0.2)	<.001

^a^ In the as-treated model, we analyzed patients in 3 groups: (1) patients allocated to the surgery group who received surgery, (2) patients allocated to the physical therapy group who completed the physical therapy protocol without having surgery during the follow-up period, and (3) patients randomized to the physical therapy group who had a delayed surgery during follow-up. We excluded patients from the as-treated analysis who were randomized to surgery but did not have surgery and those who were randomized to physical therapy but did not complete the physical therapy protocol and did not have delayed surgery.

^b^ The between-group difference at different time points and as an overall effect corrected only for International Knee Documentation Committee (IKDC) score at baseline. Positive values imply that patients did better with surgery or delayed surgery. However, none of these values indicated a clinically relevant difference.

^c^ *P* values for noninferiority based on a 1-sample *z* test with respect to the noninferiority threshold of 11 points and 1-sided α level of .025. Significant *P* values indicate that the between-group difference is significantly different with respect to the noninferiority threshold of 11 points.

^d^ Overall estimate refers to the overall IKDC score between groups including all time points.

The crude mixed-model analysis found a mean between-group difference in patient-reported knee function on the IKDC at the 5-year follow-up of 2.8 points (95% CI, −0.9 to 6.5 points; *P* < .001 for noninferiority). After adjusting for confounders, there was a mean between-group difference of 3.4 points on the IKDC questionnaire (95% CI, −0.7 to 7.4 points; *P* < .001 for noninferiority). A positive between-group value indicates greater mean improvement on the IKDC questionnaire in the surgery group compared with the physical therapy group. However, the between-group differences are significantly smaller than the noninferiority threshold of 11 points, indicating that physical therapy is not inferior to arthroscopic partial meniscectomy.

**Figure 3.**
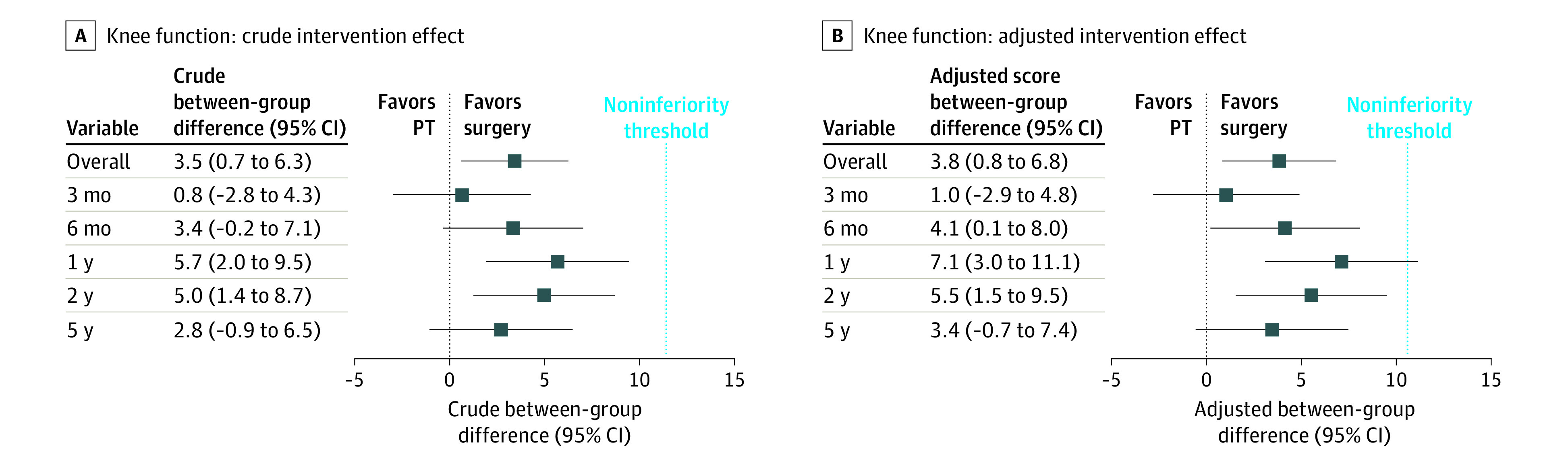
Between-Group Intervention Effects Indicated With International Knee Documentation Committee (IKDC) Subjective Knee Form Questionnaire for Physical Therapy (PT) vs Surgery Crude (A) and adjusted (B) noninferiority threshold refers to the minimal important change on the IKDC questionnaire (11 points). The squares indicate the between-group differences with 95% CIs. A positive value indicates greater improvement on the IKDC questionnaire in the arthroscopic partial meniscectomy group compared with the physical therapy group. Because none of the 95% CIs in the crude intervention effect crossed this noninferiority threshold, no clinically meaningful difference between physical therapy and surgery was observed.

Figure 3A shows crude between-group differences, and Figure 3B presents adjusted between-group differences at all follow-up time points, relative to the noninferiority threshold of 11 points on the IKDC questionnaire. Because none of the 95% CIs crossed this noninferiority threshold, no clinically meaningful difference between physical therapy and surgery was observed.

#### As-Treated Analysis

The overall crude difference between physical therapy and arthroscopic partial meniscectomy on the IKDC questionnaire score was 2.4 points (95% CI, −0.8 to 5.5 points; *P* < .001 for noninferiority) and the difference between physical therapy and delayed surgery was −3.8 points (95% CI, −8.2 to 0.6 points; *P* < .001 for noninferiority). A positive value indicates greater improvement on the IKDC questionnaire in the arthroscopic partial meniscectomy group compared with the physical therapy group, and a negative between-group value indicates greater mean improvement on the IKDC questionnaire in the physical therapy group compared with the delayed surgery group. These between-group differences were significantly different from the noninferiority threshold, indicating that physical therapy was not inferior to arthroscopic partial meniscectomy. The crude and adjusted intervention effects for all time points are presented in Table 2.

### Secondary Outcomes

#### Radiographic Knee OA

At baseline, we analyzed the radiographic images of 294 patients (surgery group, n = 146; physical therapy group, n = 148). At the 5-year follow-up, 222 radiographic images were available (surgery, n = 112; physical therapy, n = 110). We found that at 5 years of follow-up, radiographic knee OA, assessed by the OARSI sum score ranging from 0 (best) to 18 (worst), progressed by at least 1 point in 61 patients (49.2%) in the surgery group and 63 patients (50.8%) in the physical therapy group. We found a mean (SD) progression of 1.1 (2.2) points in the surgery group (from 1.9 [1.5] to 3.0 [2.6]) and 1.1 (2.1) points in the physical therapy group (from 2.1 [1.6] to 3.4 [2.7]) from baseline to 5 years. The between-group difference of 0.1 points (95% CI, −0.5 to 0.7; *P* = .78) was not significantly or clinically meaningful.

The eTable in Supplement 2 presents an overview of radiographic outcomes and patient-reported outcome measures at 5 years for the as-treated analysis. We found a progression of at least 1 point on the OARSI sum score in 52% (n = 42) of the physical therapy group, 54% (n = 61) of the surgery group, and 70% (n = 21) of the delayed-surgery group. We found no significant difference (*P* = .16) between the 3 groups in progression of the OARSI sum score from baseline to 5 years. The mean (SD) progression was 1.1 (2.2) points in the surgery group, 0.8 (2.1) points in the physical therapy group, and 1.7 (2.2) points in the delayed surgery group from a maximum of 18 points.

#### Symptomatic Knee OA

We found symptomatic knee OA in 6 patients: 4 in the surgery group and 2 in the physical therapy group. An overview of radiographic outcomes and patient-reported outcomes at 5 years can be found in the eTable in Supplement 2.

#### Additional Patient-Reported Outcomes and Surgeries

From baseline to 5 years, we found no statistically significant differences between the 2 treatment groups in pain, general physical health, and quality of life. In addition to the delayed meniscal surgeries performed in the physical therapy group (n = 52), further knee surgeries were performed in 17 patients (n = 5 surgery; n = 12 physical therapy). The eTable in Supplement 2 gives an overview of these patient-reported outcomes and additional surgeries.

## Discussion

Results of this 5-year follow-up of the ESCAPE trial showed that exercise-based physical therapy is not inferior to arthroscopic partial meniscectomy with respect to knee function during 5 years of follow-up in patients with a degenerative meniscal tear. Furthermore, we found comparable rates of progression of radiographic and symptomatic OA between both treatments.

The improvement in knee function experienced by patients in the ESCAPE trial over the first 2 years was maintained at the 5-year follow-up.^6,8^ In addition to patient-reported knee function, we found small comparable radiographic changes of the tibiofemoral joint in both treatment groups.

Our findings on patient-reported knee function are consistent with previously published trials reporting 5-year follow-up results for patient-reported knee function and pain.^12,13,14,15,16^ Our crossover rate (patients undergoing delayed surgery after initial physical therapy) of 32% was lower compared with the 38% crossover rate in the study of Katz et al^14^ but higher compared with the crossover rates of 20% reported by Berg et al^12^ and 25% reported by Sonesson et al.^16^ In our study, the as-treated results indicate that patients who received delayed surgery negatively influenced the mean knee function in the physical therapy group. In addition, after undergoing delayed surgery, the patients in the crossover group did not experience better knee function compared with those in the physical therapy group. This finding puts the added value of arthroscopic partial meniscectomy under debate, but we could not compare our as-treated results because this factor was not reported in the current literature.^12,13,14,15,16^ However, when looking at knee OA, we found 2 studies suggesting an increased risk of knee OA following surgery compared with no surgery.^14,15^ Sihvonen et al^15^ found that arthroscopic partial meniscectomy was associated with a slightly increased risk for knee OA compared with nonoperative management.^15^ Katz et al^14^ reported that patients in the surgery group had a 5 times higher risk for a total knee replacement compared with patients who only had exercise-based physical therapy.^14^ However, other trials reporting 5-year data found no significant difference in radiographic deterioration between both treatment groups.^12,13,16^ These latter findings are consistent with our results.

Furthermore, we checked for confounding effects within our primary outcomes, and our adjusted analyses are in line with our primary unadjusted results. Previous studies investigated specific patient characteristics and combinations of characteristics to estimate treatment outcome and possible subgroups of patient who will benefit more from surgery compared with physical therapy.^31,32,33,34^ However, none of these studies were able to find such a subgroup of patients. This finding is in line with ours, showing that physical therapy is noninferior to arthroscopic partial meniscectomy in patients with degenerative meniscal tears. The RCTs that reported their results on the progression of knee OA following meniscal treatment have limited power to draw conclusions that can influence clinical practice. Pooling these data using individual patient data meta-analysis will provide more reliable results. Future research should focus on pooling the 5-year data on knee function and knee OA from separate trials to strengthen clinical guidelines. In addition, investigating the effectiveness of exercise-based physical therapy compared with a wait-and-see policy or no treatment can strengthen policy makers to invest in physical therapy and enhance further deimplementation of arthroscopic partial meniscectomy for degenerative meniscal tears. Another option would be an experiment in which nonresponders to exercise therapy are randomized into a surgery group vs a radiofrequency ablation of the genicular nerve group. In patients with knee OA, radiofrequency ablation of the genicular nerve shows promising results in sham-controlled trials and may reduce the need for surgery based on pain.^35^

Other additional research should focus on facilitators and barriers for deimplementing arthroscopic partial meniscectomy. Such studies might provide insight into why so many patients opt for delayed arthroscopic partial meniscectomy following physical therapy and identify deimplementation strategies that could be tested. For instance, a natural experiment among hospitals reported that a strict evidence-based policy on knee arthroscopy in patients aged 50 years can result in a 60% decrease of arthroscopies compared with a usual-care policy.^36^ Another successful deimplementation strategy is to regulate the public financial reimbursement of knee arthroscopies.^37^

### Limitations

This study has limitations. First, during the 5-year follow-up, 52 patients (32%) from the physical therapy group underwent delayed arthroscopic partial meniscectomy—most patients (n = 44) within the first year of follow-up and only 5 patients within the last 3 years of the trial. These numbers demonstrate that not all patients experience satisfying results following physical therapy. Second, we did not register all patients’ reasons for not responding to our questionnaire or radiograph invitation. We assume that the COVID-19 pandemic would be one of the reasons why people did not attend the 5-year follow-up. Nevertheless, our response rate was high (87%). Third, radiographic sensitivity for change in knee OA is lower compared with the sensitivity of magnetic resonance imaging, and therefore magnetic resonance imaging would be preferred over radiographic images.^38^ To minimize the patient burden and study costs, we obtained radiographic images instead of magnetic resonance imaging. However, we used validated measures for OA, radiographic findings were reported by a single radiologist, and we adhered to the study protocol. A second assessor would have reduced the potential risk of observer bias in our radiographic data. However, our pragmatic approach was chosen because the radiographic data were not part of our primary outcome. This approach reflects clinical practice, and blinding the assessor strengthened the comparison between treatments. Fourth, we did not register noninvasive additional treatments for knee pain. Fifth, there is a potential risk of selection bias due to some loss to follow-up. However, we performed randomization, our missing data were equally distributed over both treatment arms (19 physical therapy and 22 arthroscopic partial meniscectomy), and the mixed-model analysis takes missing data into account using maximum likelihood estimation. Therefore, we believe it is unlikely that the missing values affected our results. Sixth, the number of patients screened for eligibility was not available.

## Conclusions

This RCT found that exercise-based physical therapy was not inferior to arthroscopic partial meniscectomy over a period of 5 years for self-reported knee function. We observed a small and comparable progression of knee OA in both groups. Findings from this trial further support the recommendation that exercise-based physical therapy should be the preferred treatment over surgery for degenerative meniscal tears.

## Notes

Supplement 1.
Study Protocol

Supplement 2. **eAppendix.** Physical Therapy Protocol **eTable.** Five-Year Outcomes for Both Treatment Groups

Supplement 3.
Data Sharing Statement
