# Supplementary material for: Effect of Physical Therapy vs Arthroscopic Partial Meniscectomy in People With Degenerative Meniscal Tears: Five-Year Follow-up of the ESCAPE Randomized Clinical Trial
Source: JAMA Netw Open. 2022 Jul 8;5(7):e2220394. doi: 10.1001/jamanetworkopen.2022.20394 (PMC9270699; doi:10.1001/jamanetworkopen.2022.20394)
Supplement: Supplement 1. — Study Protocol [file jamanetwopen-e2220394-s001.pdf]

ESCAPE protocol

"Cost-effectiveness of Early Surgery versus Conservative Treatment with Optional Delayed Meniscectomy for Patients over 45 years. A Randomized Controlled Trial.

(March 2013)

Prepared by:
drs. Victor A. van de Graaf, MD
dr. Nienke Wolterbeek, PhD
dr. Rudolf W. Poolman, MD PhD
dr. Vanessa A.B. Scholtes, PhD
dr. Camille Neeter, PhD
dr. Eduard L.A.R. Mutsaerts, MD PhD
prof. Maurits W. van Tulder, PhD
dr. Arthur de Gast, MD PhD

Dit project wordt mogelijk gemaakt door:

Version 07/ 23-9-2015

PROTOCOL TITLE '**Cost-effectiveness of Early Surgery versus Conserva-tive Treatment with Optional Delayed Meniscectomy for Patients over 45 years. A Randomized Controlled Trial. The ESCAPE trial**.'

**Protocol ID**	**NL44188.100.13 / WO11.095**
**Short title**	**ESCAPE**
**Version**	**1**
**Date**	**March 2013**
**Coordinating investigator/project leader**	***drs. V.A. van de Graaf, MD***<br>Research physician 'Onze Lieve Vrouwe Gasthuis' Amsterdam
**Principal investigators per site**	***- dr. R.W. Poolman, MD PhD***<br>Onze Lieve Vrouwe Gasthuis Amsterdam<br>***- dr. A. de Gast, MD PhD***<br>Diakonessenhuis Utrecht<br>***- dr. G.M.M.J. Kerkhoffs, MD PhD***<br>Academic Medical Center Amsterdam<br>***- drs. J. Wolkenfelt, MD***<br>Sint Lucas Andreas Ziekenhuis Amsterdam<br>***-drs. D.J. Hofstee, MD***<br>Medisch Centrum Alkmaar<br>***-dr. E.L.A.R. Mutsaerts, MD PhD***<br>Medisch Centrum Jan van Goyen Amsterdam<br>***- drs. ICJB van den Brand, MD***<br>Sint Elisabeth Ziekenhuis Tilburg<br>***- drs. D. Hoornenberg, MD***<br>Slotervaart ziekenhuis Amsterdam<br>***- dr. AMJS Vervest, MD PhD***<br>Tergooi Ziekenhuis Hilversum<br>- **prof. dr. M.W. van Tulder**<br>Supervisor economic evaluation. Dept. Health Sciences, VU University Amsterdam
**Sponsor:**<br><br>**Subsidising party**	***dr. J.Th.M. van der Schoot, chairman of Board of Management Onze Lieve Vrouwe Gasthuis Amsterdam***<br><br>**- The Netherlands Organisation for Health Research and Development (ZonMw)**

	- Stichting Achmea Gezonheidszorg
**Independent physician(s)**	***Dr. E.P. Steller, MD PhD, trauma surgeon at Sint Lucas Andreas Hospital Amsterdam***
**Laboratory sites**	***Not applicable.***
**Pharmacy**	***Not applicable.***

PROTOCOL SIGNATURE SHEET

Name	Signature	Date
**For non-commercial research,**<br>**Head of Department:**<br>Dr. E.L.A.R. Mutsaerts, MD PhD<br>Orthopaedic surgeon Onze Lieve Vrouwe Gasthuis Amsterdam		**14-3-2013**
**Coordinating Investigator/Project leader/Principal Investigator:**<br>***- dr. R.W. Poolman, MD PhD***<br>Principal investigator Onze Lieve Vrouwe Gasthuis Amsterdam<br>***Other investigators involved:***<br>***- dr. A. de Gast, MD PhD***<br>Principal investigator Diakonessenhuis Utrecht<br>***- drs. V.A. van de Graaf, MD***<br>Coordinating investigator at Onze Lieve Vrouwe Gasthuis' Amsterdam<br>***- dr. V.A.B. Scholtes, PhD***<br>Research coordinator at Onze Lieve Vrouwe Gasthuis' Amsterdam<br>***- dr. N. Wolterbeek***<br>Research coordinator at 'Sint Antonius Ziekenhuis' Nieuwegein		**14-3-2013**<br>**15-3-2013**<br>**13-3-2013**<br>**18-3-2013**<br>**18-3-2013**

TABLE OF CONTENTS

1. LIST OF PARTICIPATING CENTERS 6
2. SUMMARY 8
3. INTRODUCTION AND RATIONALE 9
4. OBJECTIVES 11
5. STUDY DESIGN 12
6. STUDY POPULATION 15
6.1 Population (base) 15
6.2 Inclusion criteria 15
6.3 Exclusion criteria 15
6.4 Sample size calculation 16
7. TREATMENT OF SUBJECTS 17
7.1 Use of co-intervention (if applicable) 17
7.2 Escape medication (if applicable) 17
8. METHODS 18
8.1 Primary and secondary outcome measures 18
8.1.1 Primary outcome 18
8.1.2 Secondary Outcomes 18
8.2 Randomization, blinding and treatment allocation 19
8.3 Study procedures 20
8.4 Study scheme 21
8.4 Withdrawal of individual subjects 22
8.5 Replacement of individual subjects after withdrawal 22
8.6 Follow-up of subjects withdrawn from treatment 22
8.7 Premature termination of the study 22
9. SAFETY REPORTING 23
9.1 Section 10 WMO event 23
9.2 Adverse and serious adverse events 23
9.3 Suspected unexpected serious adverse reactions (SUSAR) 23
9.4 Follow-up of adverse events 24
9.5 Data Safety Monitoring Board (DSMB) 24
10. STATISTICAL ANALYSIS 25
11. ETHICAL CONSIDERATIONS 27
11.1 Regulation statement 27
11.2 Recruitment and consent 27
11.3 Benefits and risks assessment, group relatedness 28
11.4 Compensation for injury 28
12. ADMINISTRATIVE ASPECTS AND PUBLICATION 29
12.1 Handling and storage of data and documents 29
12.2 Amendments 29
12.3 Annual progress report 29
12.4 End of study report 29
12.5 Public disclosure and publication policy 30
13. RISK ANALYSIS 31
14. REFERENCES 32
15. APPENDICES 34

LIST OF ABBREVIATIONS AND RELEVANT DEFINITIONS

ACL	Anterior Cruciate Ligament
ADL	Activities of Daily Living
AE	Adverse Event
APM	Arthroscopic Partial Meniscectomy
AR	Adverse Reaction
ASA	American Society of Anesthesiologists
BMI	Body Mass Index
CCMO	Central Committee on Research Involving Human Subjects; in Dutch: Centrale Commissie Mensgebonden Onderzoek
CV	Curriculum Vitae
DSMB	Data Safety Monitoring Board
EudraCT	European drug regulatory affairs Clinical Trials
GCP	Good Clinical Practice
HLQ	Health and Labour Questionnaire
IC	Informed Consent
ICRS	International Cartilage Repair Society
IKCD	International Knee Documentation Committee
ISAKOS	International Society of Arthroscopy, Knee Surgery and Orthopedic Sports Medicine
KOOS	Knee injury and Osteoarthritis Outcome Score
MREC	Medical research ethics committee (MREC); in Dutch: Medisch Ethische Toetsing Commissie (METC)
MRI	Magnetic Resonance Imaging
OA	OsteoArthritis
PSC	Patient Specific Complaints (in Dutch: Patiënt Specifieke Klachten)
PE	Physical Examination
QOL	Knee-related Quality Of Life
(S)AE	(Serious) Adverse Event
SPC	Summary of Product Characteristics (in Dutch: officiële productinfomatie IB1-tekst)
SUSAR	Suspected Unexpected Serious Adverse Reaction
Wbp	Personal Data Protection Act (in Dutch: Wet Bescherming Persoonsgevens)
TiC-P	Trimbos/iMTA questionnaire for Costs associated with Psychiatric Illness (TiC-P)
VAS	Visual Analogue Scale
WMO	Medical Research Involving Human Subjects Act (in Dutch: Wet Medisch-wetenschappelijk Onderzoek met Mensen

1. LIST OF PARTICIPATING CENTERS

1. Onze Lieve Vrouwe Gasthuis
Hoofdonderzoeker: V.A. van de Graaf, MD
Verantwoordelijk staflid: R.W. Poolman, MD PhD
Onze Lieve Vrouwe Gasthuis
Postbus 95500
1090 HM Amsterdam
Tel: 020 - 5993050

2. Diakonessenhuis
Verantwoordelijk staflid: A. de Gast, MD PhD
Diakonessenhuis Utrecht
Postbus 80250
3508 TG Utrecht
Tel: 088 - 2506912

3. Sint Lucas Andreas Ziekenhuis
Verantwoordelijk staflid: J. Wolkenfelt, MD
Sint Lucas Andreas Ziekenhuis
Postbus 9243
1006 AE Amsterdam
Tel: 020 – 5108911

4. Academisch Medisch Centrum
Verantwoordelijk staflid: G.M.M.J. Kerkhoffs, MD PhD
Academisch Medisch Centrum
Postbus 22660
1100 DD Amsterdam
Tel: 020 - 5669111

5. Medisch Centrum Alkmaar
Verantwoordelijk staflid: D.J. Hofstee, MD
Medisch Centrum Alkmaar
Postbus 501
1800 AM Alkmaar
Tel: 072 – 5484444

6. Medisch Centrum Jan van Goyen
Verantwoordelijk staflid: E.L.A.R. Mutsaerts, MD PhD
Medisch Centrum Jan van Goyen
Jan van Goyenkade 1
1075 HN Amsterdam
Tel: 020 – 3055800

7. Sint Elisabeth Ziekenhuis
Verantwoordelijk staflid: I.C.J.B. van den Brand, MD
Sints Elisabeth Ziekenhuis
Hilvarenbeekse Weg 60
5022 GC Tilburg
Tel: 013 – 5391313

8. Slotervaart Ziekenhuis Amsterdam
Verantwoordelijk staflid: D Hoornenberg, MD
Slotervaart Ziekenhuis
Louwesweg 6
1006 BK Amsterdam
Tel: 020 – 5129333

9. Tergooi Ziekenhuis Hilversum
Verantwoordelijk staflid: A.M.J.S. Vervest MD PhD
Tergooi Ziekenhuis
Van Riebeeckweg 212
1213 XZ Hilversum
Tel: 088 – 7351735

10 Medisch Centrum Haaglanden
Verantwoordelijk Staflid: E.R.A. van Arkel MD
Medisch Centrum Haaglanden
Lijnbaan 32
2512 VA Den Haag
Tel: 070 - 357 44 44

2. SUMMARY

Rationale: Current standard treatment of symptomatic non-obstructive meniscal tears in older patients is surgery. Annual costs are 33 million Euros in this patient group (N=15.000) in the Netherlands. Nevertheless, evidence is lacking that supports its superiority over conservative treatment. When conservative treatment is non-inferior to surgery, this strategy alone could save over 12 million Euros on an annual basis.
We therefore risk large healthcare inefficiency, since these patients are treated surgically. The financial benefits of conservative treatment might even be enhanced by an anticipated decrease in the progression to knee osteoarthritis, since fewer knee arthroplasties would be necessary. This could even further decrease the annual costs spent on knee surgeries.

Objective: This multicenter randomized controlled trial is designed to compare surgical to conservative treatment of non-obstructive meniscal injuries in older patients.

Hypothesis: We assume equal improvement of physical function in both groups and reduced costs with conservative treatment.

Study design: Non-inferiority multicenter randomized controlled trial with an economic evaluation alongside. The study will be conducted by the Orthopaedic Research Consortium Mid-West Netherlands and performed in 9 clinics, including 1 academic medical center.

Study population: We will include 320 patients between 45 and 70 years with MRI-confirmed symptomatic, non-obstructive meniscal tears. Two groups160 patients, are needed to prove non-inferiority of conservative therapy. Block randomization will be done web-based.

Measurements: Patients will be asked to complete questionnaires at baseline and 3, 6, 9, 12, 18 and 24 months. At 3 months they will visit the outpatient department for physical examination. At 24 months an X-ray will be obtained. We also plan a follow-up at 60 months.

Primary outcome: Physical function, measured by International Knee Documentation Committee ‘Subjective Knee Form’.

Secondary outcome: general health (RAND-36), quality of life (EQ-5D5L), level of activity (Tegner Activity Scale), knee pain on Visual Analogue Scale (VAS), productivity losses and the use of healthcare services (TiC-P), patient specific complaints (PSC), a patient’s expectation and their satisfaction of treatment, physical examination, progression of osteoarthritis and the occurrence of adverse events.

Cost-effectiveness and Budget Impact Analysis: We will perform a cost-effectiveness and cost-utility analysis from societal perspective. For this we will measure productivity losses and the use of healthcare services. All relevant costs will be measured, valued and analyzed. Cost-effectiveness ratios and planes will be established using bootstrapping techniques (5000 replications).
A Budget Impact Analysis will be performed from societal, government and insurer perspective.

Extent of burden: In this multicenter randomized controlled trial, the current standard treatment, surgery, will be compared to conservative treatment, consisting of 16 sessions of structured physical therapy. Physical therapy is a safe treatment and is not known with any risks or complications. Since patients from the conservative group will be able to undergo delayed surgery when conservative treatment has failed, we are convinced this study is safe and without any additional risks.

3. INTRODUCTION AND RATIONALE

Probleemstelling / Problem definition

Meniscal surgery is the most performed orthopaedic surgical intervention with 30,000 procedures annually (1). In the U.S. there was a 49% increase in arthroscopic partial meniscectomies (APM) between 1996 en 2006 (2). Half of these were performed in patients over 45 years old. These numbers continue to rise since the proportion of population over 60 years will double from 11% to 22% between 2000 and 2050 (WHO). APM therefore contributes significantly to the costs of our health care system.

APM is the current treatment of choice for meniscal tears. When symptoms persist or in case of mechanical obstruction (locking and limited range of motion) APM has been proven to be an effective treatment to restore knee function (1;3). In older patients when mechanical obstruction is usually absent, it is unclear whether surgery is superior over conservative treatment to reduce symptoms.

Despite the wide use of APM for treatment of non-obstructive meniscal lesions, Randomized Controlled Trials (RCTs) on this subject are sparse. Howell (2009) (4) described in a Cochrane review that no conclusions could be drawn on a favorite role of surgery or conservative treatment due to a lack of RCTs. However, this review is out-of date and currently being updated by Mutsaerts et al. (5).

We have found only 4 RCTs (3;6-8) that compared surgery with conservative treatment in patients with meniscal tears. All these studies found no difference in outcome between groups. However, the power of these studies was too small to establish or rule out a true difference in effectiveness and the heterogeneity between the studies was too large to pool the data. It therefore remains unclear whether APM is more effective compared to conservative treatment on functional outcome.

Although arthroscopy for obstructive meniscal tears is widely accepted, non-obstructive complaints may not be triggered by meniscal tears, but by early onset osteoarthritis (OA) in older patients. This is strongly supported by a study of Englund (2008) (9) who identified meniscal tears on MRI in 61% of nearly 1000 asymptomatic volunteers over 50 years old. Treatment of non-obstructive meniscal tears focuses on reducing symptoms, while the meniscal tear itself might be asymptomatic and APM only reduces its function.

Furthermore, wide-spread utilization of arthroscopy in patients with knee OA came under scrutiny since two publications in the New England Journal of Medicine on arthroscopy for OA showed no benefit from arthroscopy over sham surgery (10;11).

Since quality of the menisci decreases with ageing and they become more vulnerable to damage and tears (12-14), both surgery and conservative treatment may not prevent the development of OA. However, APM in degenerative knees may accelerate this process more than a non-operative approach. This may therefore influence the number of knee arthroplasties subsequently needed. Faster progression to OA will lead to more patients on waiting lists for knee replacement an subsequently raise costs. In 2003, the National Hospital Discharge Survey in the U.S. described a total of 402,100 knee arthroplasties in 2003 and predicted this to grow with 673% to 3.48 million by 2030 (15), indicating its relevance. Preventing the acceleration of OA may result in stagnation of these numbers. A substantial reduction of costs of healthcare utilization could be accomplished.

Lastly, reducing the number of surgeries may result in less iatrogenic damage to cartilage in the knee and less adverse events related to surgery, which also leads to an improvement of patient outcomes and a reduction of costs.

A meniscal tear could thus lead to knee OA, but knee OA could also lead to a spontaneous meniscal tear (16).

We therefore hypothesize meniscal tears not as predominant factor causing knee symptoms in patients over 45 years and see an opportunity for conservative treatment.

HEALTH CARE EFFICIENCY PROBLEM:

There is thus a large health care efficiency problem as in older patients with non-obstructive symptomatic meniscal tears, expensive surgery is widely applied though there is no evidence that this approach is more effective compared with less costly conservative treatment.

RELEVANCE FOR PRACTICE:
The present study proposal is relevant for several reasons.

1. Meniscal surgery is the most performed orthopaedic intervention.
   Each year approximately 30.000 meniscal surgeries are performed in the Netherlands and half of these are performed in patients over 45 years old. Surgery is the current standard therapy for this patient group. With ageing of the population and the recent changes of reimbursement of physical therapy, it is expected that the number of meniscal surgeries will only increase further.

2. The standard therapy is performed despite a lack of evidence in times of evidence-based medicine.

3. Potential cost savings with the rising costs of health care system.
   As mentioned above, 15,000 meniscal surgeries are performed annually in the Netherlands in patients over 45 years. We estimate that 2/3 of surgeries can be prevented, which besides an estimated cost reduction of €12 million each year leads to less iatrogenic damage and fewer adverse events.

4. Investigator initiated research.
   Lexchin et al. (2003) (17) showed that pharmacy involvement in or sponsoring of clinical trials clearly influences research outcome, indicating there is a strong need for independent investigator initiated studies. This investigator initiated trial has, despite of possible negative consequences for surgeons in terms of fewer surgical procedures, only one aim in optimizing a patient’s quality of life.

5. Progression of osteoarthritis.
   We are uninformed about the progression of OA after meniscal surgery. A reduction of surgery for meniscal tears in patients with OA might reduce the progression of OA, which could lead to a stagnation of patients on waiting lists for total knee replacement.

4. OBJECTIVES

The objective of this study is to evaluate the effectiveness and cost-effectiveness of surgical and conservative treatment of non-obstructive meniscal injuries in older patients

Hypothesis:
We assume equal improvement of physical function in both groups and reduced costs with conservative treatment.

5. STUDY DESIGN

We plan a non-inferiority multicenter RCT with an economic evaluation alongside. The study conducted by the Orthopaedic Research Consortium Mid-West Netherlands. Patients will be randomized into 2 equal groups of 160 patients and will receive either APM or PT.

PRIMARY OUTCOME:
Primary outcome will be change in physical function from baseline to 2 years measured by the International Knee Documentation Committee (IKDC) 'Subjective Knee Form', which has been validated for meniscal injuries (18).
In addition, we will perform an economic analysis alongside the RCT from a societal perspective and a budget impact analysis from societal, government and insurer perspective.

SECONDARY OUTCOMES:
1) Change in:
- General health, measured by RAND-36;
- Quality of life, measured by EQ-5D5L;
- Pain, measured with the VAS both in rest and weight bearing;
- Level of activity, measured by Tegner Activity Scale (TAS);
- Patient specific complaints measured by the PSC (patient specific complaints) Questionnaire;
- Treatment group; number of patients initially treated conservatively, treated secondarily by APM.

We will use the EQ-5D5L to measure Quality Adjusted Life Years (QALY).
2) Productivity losses and the use of healthcare services, measured by a modified version of the Trimbos/iMTA questionnaire for Costs associated with Psychiatric Illness (TiC-P).
3) Relation between a participants expectation of treatment and their satisfaction.
4) Physical Examination (PE), consisting of performance on physical tests (squatting with duckwalk, Thessaly test, McMurray), the range of motion, joint line tenderness and the existence of joint effusion in the knee.
5) Adverse events including:
-Minor: prolonged synovial fluid leakage from arthroscopy portals and bleeding
-Moderate: surgical site infection, vascular and neurological damage
-Severe: septic arthritis, cardiac events, pulmonary embolism and death
Surgical instrument malfunction will be recorded, as well as reoperations including knee arthroplasties and re-hospitalization.

6) We plan a follow up moment at 60 months to see the progression of osteoarthritis, measured with the Kellgren Lawrence Grading Scale for Osteoarthritis (appendix C).

X-ray and MRI will both be made at baseline, X-ray will be repeated after 2 and 5 years of follow up. All MRI’s will be evaluated by describing the quality of the meniscus based upon signal intensity and the type of tear; longitudinal, horizontal or radial. All other structures, especially the other ligaments in the knee, will be judged as well. This will be done experienced radiologist, specialized into musculoskeletal radiology.

In order to get compatible results, randomization will be stratified for age. The activity level will also be assessed at baseline. This will be done by the Tegner scale and Activity Rating Scale (ARS).

FOLLOW-UP:
Patients will visit the outpatient department for physical examination (3 months) and X-rays (24 months). All measurement outcomes will be obtained at baseline, 3, 6, 9, 12, 18 and 24 months, online or by hardcopy if preferable. Patients will be examined by a well experienced clinician at each site. The anticipated enrolment period will be approximately one year with follow up.
We also plan to perform another long-term outcome measurement after 5 years.

Endpoint: the first results of the study will be evaluated after 1 year of follow up. The endpoint for the primary outcome of the study will be after 2 years of follow up.
However, we plan a follow up moment at 60 months to see the progression of osteoarthritis, measured with the Kellgren Lawrence Grading Scale for Osteoarthritis.

Sample size calculation:
Sample size is based on a power of 90%, an alpha of 0.05, a standard deviation of 18 points and a non-inferiority threshold of 8 points on the IKDC 'Subjective Knee Form'.
We calculated that with 10% loss to follow-up after 24 months and 25% delayed APM in PT group, 160 patients are needed per group in this equivalence type RCT. This means a total of 320 patients will be included. Sample size is calculated for the intention-to-treat analysis.

Assigning patients to treatment groups:
After signing and dating the informed consent form, patients will be randomized. This will be done in a web-based system, using the ALEA software, stratified for each site and for 2 age subgroups:

Subgroup 1: 45-57 years of age
Subgroup 2: 58-70 years of age

Patients who are excluded from the study will get treatment at the discretion of the surgeon. Patients who prefer not to participate will be asked for their reasons. These reasons will be recorded.

Data Management:
Database management will be done using a web-based system.

Safety: the occurrence of adverse events and subsequent knee surgeries will be queried and recorded at follow-up visits.

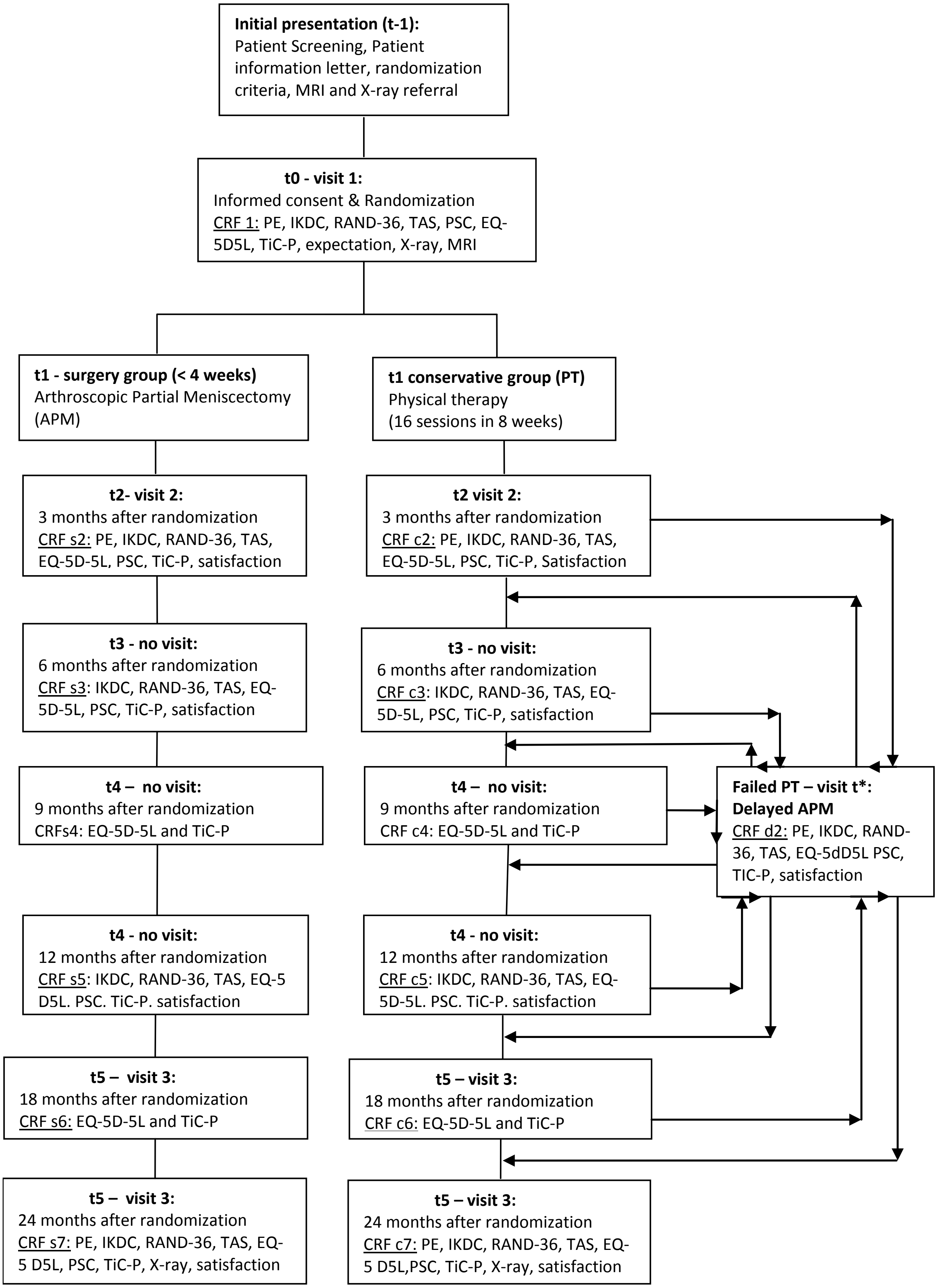

Figure 1: Study Flowchart

* Total follow up time for primary outcome in delayed APM group remains 24 months

CRF s* = surgery / CRF c*=conservative, CRF d*=delayed

6. STUDY POPULATION

6.1 Population (base)

STUDY POPULATION:
We will include 320 patients between 45 and 70 years with symptomatic, non-obstructive MRI confirmed meniscal tears. We will study female and male patients of different ethnic backgrounds. Block randomization and database management will be done using a web-based system.

6.2 Inclusion criteria

1) Patients between 45 and 70 years of age at presentation.
2) A meniscal tear visualized on MRI. The meniscal tear can either be isolated or combined with a partial asymptomatic Anterior Cruciate Ligament (ACL) injury or a asymptomatic degenerative ACL shown on MRI with no abnormal clinical findings (a negative Lachman test and Pivot Shift).
3) Mental Competence.
4) Willingness to comply with follow-up schedule.
5) Written informed consent.

6.3 Exclusion criteria

6) Knee locking or trauma leading to acute surgery.
7) One of the following associated injuries on the index knee:
   a. A symptomatic partial ACL rupture or any total ACL rupture determined by clinical examination (positive Lachman test and/or positive Pivot Shift) and shown on MRI;
   b. A complete PCL injury;
   c. Cartilage change down to bone; grade 4 of the Kellgren Lawrence Grading Scale for Osteoarthritis visualized on X-ray;
   d. An injury to the lateral/posterolateral ligament complex with significantly increased laxity.
8) A history of knee surgery other than diagnostic arthroscopy on the index knee.
9) Tumors on MRI suspected for a malignancy.
10) Obese patients with BMI > 35.
11) ASA 4-5 (appendix D) patients which can interfere with revalidation.
12) General disease that effects physical function or systemic medication/abuse of steroids (e.g., rheumatoid arthritis, psoriatic arthritis, systemic lupus erythematosus, gout, pseudogout)
13) Any other medical condition or treatment interfering with the completion or assessment of the trial, e.g. contraindications to MRI or surgery.
14) Drugs or alcohol abuse.
15) Patients unable to speak or read Dutch.

Nature and extent of the burden and risks associated with participation, benefit and group relatedness:
At baseline, when a meniscal tear is suspected at presentation, an MRI-scan will be obtained. This is not seen as an extra burden for participants, since an MRI which has a high sensitivity and specificity normally is usually made to avoid unnecessary diagnostic arthroscopies as stated in the guideline for arthroscopy of the knee, 2010 (NOV) (1).
All patients approached and willing to participate in the trial will be randomized when all inclusion criteria are met when they return to the outpatient department for the results of the MRI.
Participants will be randomized into the surgical group or the conservative group and are followed at 3, 6, 9, 12, 18 and 24 months.

The risks associated with participation in the trial include the general risks for surgery, e.g. infection, bleeding, postoperative numbness, paralysis, pain, persistence of symptoms when randomized into the surgical group. In the conservative group, no specific risks are known to occur. Since surgery is current standard treatment, there are no additional risks for participation in this trial.
All patients will return to the outpatient department after 3 and 24 months. This will be for PE (3 months) and X-rays (24 months).
We plan a follow up moment at 60 months to see the progression of osteoarthritis, measured with the Kellgren Lawrence Grading Scale for Osteoarthritis.
The burden of the extra X-ray will be negligible, especially when focused on the potential outcome of the trial, which could lead to a significant reduction of unnecessary surgeries.
All questionnaires will be obtained digitally and can be completed at home. If preferred, a hardcopy can be sent to these participants.
Completion of the questionnaires will take approximately 30 minutes. The questionnaires at 9 and 18 months consist only of the EQ-5D-5L and TiC-P and will take only 5 minutes to complete.

6.4 Sample size calculation

Sample size is based on a power of 90%, an alpha of 0.05, a standard deviation of 18 points and a non-inferiority threshold of 8 points on the IKDC (a minimal clinical important change in IKDC is 8.8) (18).
We calculated that with 10% loss to follow-up after 24 months and 25% delayed APM in the PT group, 160 patients are needed per group in this non-inferiority RCT.

This corresponds to a total of 320 patients will be included.

FEASIBILITY:
In this multicenter randomized trial, performed by the Orthopaedic Consortium Mid-West Netherlands, nine centers participate with large volumes of meniscectomies (over 4000 meniscectomies annually). The Onze Lieve Vrouwe Gasthuis Amsterdam, Academic Medical Center Amsterdam, Diakonessenhuis Utrecht, the Sint Lucas Andreas Ziekenhuis Amsterdam, the Medisch centrum Jan van Goyen, the Medisch Centrum Alkmaar, the Sint Elisabeth Ziekenhuis Tilburg, the Slotervaart Ziekenhuis Amsterdam, Medisch centrum Haaglanden and the Tergooi ziekenhuis Hilversum. Despite age criteria and exclusion of mechanical obstruction, we expect a high recruitment rate, since patients will not be refrained from surgery in the long run. Assuming a participation of 30-40%, we therefore expect that inclusion will be completed within 27 months and all patients have finished their follow-up no later than 48 months after the start of the study.

7. TREATMENT OF SUBJECTS

Intervention group:

Arthroscopic partial APM is performed within 4 weeks in day-care by an (orthopaedic) surgeon experienced in arthroscopic surgery, or by a resident skilled in arthroscopic knee surgery under direct supervision of an orthopaedic surgeon experienced in arthroscopic surgery. Eight weeks after surgery (3 months after randomization), patients will visit the outpatient department to check for function and persistence of symptoms. Since standard PT after APM has not been proven effective, patients will only be referred to a physical therapist in case of swelling or signs of atrophy, as advised by the Dutch Orthopaedic Association Guidelines (1).

Control group:

PT consists of 2 sessions of 30 minutes per week for 8 weeks, with a total of 16 sessions. Patients will also be given a home exercise program (appendix B). These programs are based on a physical therapy program used by Herrlin et al. (6) and is adjusted for our population by a physical therapist (dr. C. Neeter; member of the research group) who earned his PhD degree in the field of the anterior cruciate ligament and is specialized on the knee.
After completion of the PT-sessions, patients will visit the outpatient department to check for function and persistence of symptoms.

Delayed surgery group:

Based on patients complaints, findings during physical examination and the level of pain, the physician and/or surgeon and patient will decide in agreement that conservative treatment has failed and choose for delayed arthroscopic partial meniscectomy. This can be done no earlier than 3 months after randomization during the entire follow up time of the study.
Prior to the delayed APM patients will be asked to answer an extra questionnaire as endpoint of PT. Patients in this group will be analyzed in the PT group according to the Intention-to-treat principle.

7.1 Use of co-intervention (if applicable)

Medicational use, in terms of analgetics are allowed to use during the study duration.

Patients will be asked for the use of analgetics during the study.

7.2 Escape medication (if applicable)

Not applicable.

8. METHODS

8.1 Primary and secondary outcome measures

8.1.1 Primary outcome

Primary outcome will be change in physical function from baseline to 2 years measured by the International Knee Documentation Committee (IKDC) 'Subjective Knee Form', which has been validated for meniscal injuries (18). The IKDC is developed for knee-specific measurement of symptoms, function, and sports activities in patients with ligament and meniscal injuries. The IKDC is a self-administered questionnaire with a total of 19 questions. All items, except item 10a, are converted to a score with a maximum of 100 indicating no restrictions in daily and sports activities and the absence of symptoms. A difference of more than 8.8 points in IKDC-score is deemed clinically relevant.
In addition, we will perform an economic analysis alongside the RCT from a societal perspective and a budget impact analysis from societal, government and insurer perspective.

8.1.2 Secondary Outcomes

1) Change in:
- General health, measured by RAND-36
- Quality of life, measured by EQ-5D5L
- Pain, measured with the VAS in rest and during weight bearing.
- Level of activity, measured by Tegner Activity Scale (TAS)
- Patient specific complaints measured by the PSC (patient specific complaints) Questionnaire
- Treatment group; number of patients initially treated conservatively, treated secondarily by APM.

We will use the EQ-5D5L to measure Quality Adjusted Life Years (QALY).
2) Productivity losses and the use of healthcare services, measured by a modified version of the Trimbos/iMTA questionnaire for Costs associated with Psychiatric Illness (TiC-P).
3) Relation between a participants expectation of treatment and their satisfaction.
4) PE, consisting of performance on physical tests (squatting with duckwalk, Thessaly test, McMurray), the range of motion, joint line tenderness and the existence of joint effusion in the knee.
5) Adverse events including:
- Minor: prolonged synovial fluid leakage from arthroscopy portals and bleeding.
- Moderate: surgical site infection, vascular and neurological damage
- Severe: septic arthritis, cardiac events, pulmonary embolism and death.

Surgical instrument malfunction will be recorded, as well as reoperations including knee arthroplasties and re-hospitalization.

6) We plan a follow up moment at 60 months to see the progression of osteoarthritis, measured with the Kellgren Lawrence Grading Scale for Osteoarthritis.

Hypotheses:
1) We assume equal improvement of physical function in both groups (a difference of < 8.8 points on IKDC ‘Subjective Knee Form’). This would question the role of arthroscopic surgery in treatment of symptomatic, non-obstructive meniscal injuries in patients over 45 years old.
2) We assume with an equal improvement of function in both groups, that an initial conservative approach for this condition in older patients is much more cost-effective than APM as standard therapy.
3) We assume a lower baseline value of participants compared to the average score of the Dutch population and an equal improvement of general health in both groups (RAND-36).
4) We assume equal improvement of quality in life, measured in QALY’s.
5) We assume equal improvement of pain, measured with the VAS score.
6) We assume equal improvement of activity level (Tegner Activity Score).

7) We assume equal improvement of complaints in activities and movements found most important by patients (patient specific complaints).
8) We expect a greater reduction of joint line tenderness in the arthroscopic partial meniscectomy group shortly after therapy, compared to the physical therapy group, since part of the damaged meniscus is released. However, we expect no difference in joint line tenderness on the long term. We expect a faster reduction of joint effusion after treatment in the physical therapy group. However, we do not expect a difference between both groups after 24 months. We do not expect a significant improvement of ROM after treatment in both groups.
9) We estimate that 2/3 of surgeries can be prevented (as stated by Herrlin SV et al. (6), and therefore expect 1/3 of patients who will undergo delayed arthroscopic meniscectomy after completion of physical therapy. The possible cost reduction of this effect will be calculated in the economic analysis.
10) Patients in the physical therapy group will leave work for at least 16 sessions of 30 minutes each week, representing one working day, with the addition of travelling time. Patient in the surgery group are expected to be absent from work for one working day, the day of surgery. We therefore expect surgery to lead to more productivity losses.
11) We expect squatting to have the greatest predictive value of the different meniscal tests for detecting meniscal injuries.
12) We expect a faster development or progression of osteoarthritis after arthroscopic partial meniscectomy compared to physical therapy.
13) We expect adverse events to occur more frequently in the arthroscopic partial meniscectomy group. Patients who are subjected to delayed surgery after physical therapy has failed, remain in the physical therapy group. Therefore, we do expect adverse events to occur in the physical therapy group. Since the number in the arthroscopic partial meniscectomy group are to be expected to outnumber the number of patients with delayed surgery, we expect more adverse events in the arthroscopic partial meniscectomy group.

8.2 Randomization, blinding and treatment allocation

We will include 320 patients between 45 and 70 years with MRI-confirmed symptomatic, non-obstructive meniscal tears. Randomization will be performed in a 1:1 ratio by a computerized software program (TENALEA Clinical Trial Data Management System) using random blocks with maximum block size 6, stratified for center and age group.

a. Open study (no blinding)
b. Number of randomization arms : 2
c. Specification of the arms : APM vs PT
d. Weight of the arms : 1:1
    - Type of randomization : Random block, max block size 6
e. Strata and categories:
    - Age : (45-57) and (57-70)
    - Centers : a maximum of 10
f. No deletion or replacement of participants (unless false randomization due to clear technical issues)

At time of presentation at the outpatient department, people suspected for a meniscal injury will be informed about the study with an information letter. When they return to the outpatient department for the results of the MRI, they will be asked for participation in the study. After informed consent is signed, the local physician/surgeon contacts the randomization website and submits a patient’s details and treatment center to the online TENALEA data management program and receives treatment allocation when submitting this information to the website. All data management will be recorded online, unless patients are unable or unwilling to complete the questionnaires online.

8.3 Study procedures

a) APM group: Arthroscopic partial meniscectomy will be performed in patients with an MRI confirmed meniscal injury. The meniscectomy will be performed within 4 weeks after randomization. During the arthroscopic surgery, the different joint components, cruciate ligaments, collateral ligaments and the cartilage will be evaluated. A (orthopaedic) surgeon, experienced in arthroscopic surgery will perform meniscectomy. During this procedure, he will trim the damaged meniscus back to a stable rim. Loose fragments of cartilage and bone can be trimmed as well for a smooth surface, but they do not attempt to stimulate a healing response by, such as microfracturing. Intraarticular corticosteroid injections were not permitted at the time of surgery. Preoperative antibiotics were used routinely, according to the standard protocol at the site.

b) Conservative group: A physical therapy program was developed by a knee specialized and PhD physical therapist (dr. C. Neeter, member of the project group) for this study (Appendix B). With permission of prof. SV Herrlin, the protocol of her study was used and adapted based on evidence in the literature supporting land-based, individualized physical therapy with concomitant progressive home-exercise for patients with kneeOA. The active rehabilitation program is designed around cardiovascular (circulation), coordination and balance, and closed-chain strength exercises. Shearing forces in the knee are less using closed-chained exercises compared to open-chained exercise. Close-chained exercise activates both agonists and antagonists around the knee joint resulting in a direct rotatory movement and prevents from shearing forces seen by open-chained exercises. Heijne studied the role of open- and close-exercise in the rehabilitation after a reconstruction of the anterior cruciate ligament and advised to be careful with open-chained exercises in the early start of rehabilitation (19;20). The program consists of 16 session of physical therapy, 2 sessions per week. Besides, a written home exercise program will be given to the participants of this group (Appendix B).

c) *MRI:* an MRI will be made at baseline to confirm the diagnosis of a meniscal tear. A radiologist, experienced in musculoskeletal radiology will judge the MRI. All MRI's will be evaluated by describing the quality of the meniscus based upon signal intensity, type of lesion; a traumatic or degenerative tear, and a horizontal or vertical tear. All other structures, such as the synovium, subchondral bone, ligaments, bursae and cartilage, will be judged as well.

d) *X-ray:* an X-ray will be made at baseline and at 24 of follow up. A radiologist, experienced in musculoskeletal radiology will judge the X-rays. The joint space will be measured in all components corresponding to their narrowest part. We also plan a follow up moment at 5 years to identify the development or progression of OA.

e) *IKDC:* The International Knee Documentation Committee developed the 'Subjective Knee Form' (2006). It was developed for knee-specific measurement of symptoms, function, and sports activities in patients with a variety of knee conditions, including ligament and meniscal injuries, articular cartilage lesions, and patellofemoral pain. The IKDC is a self-administered questionnaire with a total of 19 questions. Response options include dichotomous, 11-point numeric rating scales and 5-point Likert scales. All items, except item 10a, are converted to a score with a maximum of 100 indicating no restrictions in daily and sports activities and the absence of symptoms. The questionnaire takes approximately 5 minutes to complete. The IKDC 'Subjective Knee Form' has been validated for meniscal injuries by Crawford et al. 2007 (18) and was translated and validated in Dutch by Haverkamp D et al. in 2008 (21).

f) The RAND-36 is a self-administered questionnaire and measures general health status. It consists of eight dimensions of health, including: Physical function, Role limitations due to Physical and Emotional problems, Bodily pain, General health, Vitality, Social function and Mental health. The RAND-36 has a total of 36 questions and the overall score varies between 0-100, where a higher score indicates a better function. Also, two aggregated scores can be calculated, based on the average scores of the Dutch population. These scores are the Physical and Mental component score and the averages have been set on 50. The RAND-36 takes approximately 10 minutes to complete.

g) *EQ-5D5L - Quality of Life:* The generic effects on quality of life will be assessed with the Euroqol EQ-5D5L (22). This widely used quality-of-life instrument includes five dimensions

of health related quality of life, namely mobility, self-care, daily activities, pain/discomfort, and depression/anxiety. These five dimensions will be combined into a health state.

h) *Costs and EQ-5D5L - Cost-utility*: To establish the costs, relevant cost items are identified, after which these costs are measured and values are placed on the cost items. These relevant cost items are: the total surgery time and the costs surgery versus the costs of physical therapy.
   Utility values will be calculated for the health states of the EQ-5D5L, using preferences elicited from a general Dutch population. The utility values will be used to compute quality-adjusted life-years (QALY-EQ-5L5D) by means of the area under the curve method.
i) *TiC-P part II* (*Trimbos/iMTA questionnaire for Costs associated with psychiatric illness)* measures costs and consequences of productivity losses (indirect costs). Part II of the TiC-P is a shortened version of the Health and Labour questionnaire and is appropriate for the measurement of productivity losses in both physical and psychiatric conditions. It has 4 modules: absenteeism from paid work, production losses without absenteeism from paid work, unpaid work and nuisance in paid and unpaid work and contains of 11 questions (23). In this modified version of the TiC-P, prepared by prof. M. van Tulder of the VU University Amsterdam, we will also measure the use of healthcare services.
j) *PSC (patient specific complaints)* is a patient reported questionnaire, in which patients report on specific activities and movements in which they experience symptoms. It contains of 28 normal daily activities and movements. The patient selects 3 activities or movements in which they experience symptoms and they prefer to improve in the next months. They are then asked to fill in a visual analogue scale (of 10 cm) in which report the amount of complaints they experience (24).
k) *Tegner activity scale* is a numerical scale ranging from 0 to 10 (25). Each value indicates the ability to perform specific activities. An activity level of 10 corresponds to participation in competitive sports, including soccer, football, and rugby at the elite level; an activity level of 6 points corresponds to participation in recreational sports; and an activity level of 0 is assigned if a person is on sick leave or receiving a disability pension because of knee problems. An activity level of 5 to 10 is recorded only if the patient participates in recreational or competitive sports. The psychometric were tested by Briggs KK. et al. 2006 (26) and were found acceptable for patients with a meniscal injury of the knee.

8.4 Study scheme

At the initial presentation, an MRI-scan will be obtained when a meniscal injury is suspected. Patient will also be informed about the trial and will be given a patient information latter. In case a meniscal injury is diagnosed, patients will be asked to participate in the trial.

After informed consent is signed at the outpatient department (t0), patients will be entered in an online randomization program and are assigned to either arthroscopic partial meniscectomy (within 4 weeks) or physical therapy. They are asked to complete CRF t0. 3 months after treatment, they will return to the outpatient department to check for function. A physical examination is performed, and they will be asked to complete CRF t2. At both 6 (t3), and 12 (t4) months they will not visit the outpatient department and they are online asked to complete the corresponding CRF. For an overview of assessments, see figure 1 or table 1. At 24 months they will be asked to visit the outpatient department for an X-ray and to complete CRF t5 as endpoint of the study.

In case patients in the physical therapy group don’t show any improvement, they will be held the choice of delayed arthroscopic partial meniscectomy. This will be determined by agreement between participant and surgeon. Each CRF is calculated to take no more than 30 minutes.

Table 1: measurement moments

Baseline (t0)	1-2 months (t1)	3 months (t2)	6 months (t3)	9 months (t4)	12 months (t5)	18 months (t6)	24 months (t7)
CRF-1		CRF-2	CRF-3	CRF-4	CRF-5	CRF-6	CRF-6
Visit 1	Treatment	Visit 2	No visit	No visit	No visit	No visit	visit 3
Randomization	APM or PT						
IKDC		IKDC	IKDC		IKDC		IKDC
RAND-36		RAND-36	RAND-36		RAND-36		RAND-36
EQ-5F5L		EQ-5D5L	EQ-5D5L	EQ-5D5L	EQ-5D5L	EQ-5D5L	EQ-5D5L
VAS		VAS	VAS		VAS		VAS
TAS		TAS	TAS		TAS		TAS
PSC		PSC	PSC		PSC		PSC
TiC-P		TiC-P	TiC-P	TiC-P	TiC-P	TiC-P	TiC-P
PSC		PSC	PSC		PSC		PSC
PE		PE					
X-ray							X-ray
Expectation		Satisfaction	Satisfaction		Satisfaction		Satisfaction
MRI							

8.4 Withdrawal of individual subjects

Participants are free to leave the study at any time for any reason if they wish to do so without any consequences. The investigator can also decide to withdraw a subject from the study for urgent medical reasons.

Participants who leave the study, will be considered as a drop-out and will be contacted in order to obtain information about the reasons for this and will be checked for any adverse events.

8.5 Replacement of individual subjects after withdrawal

Patients will not be replaced after withdrawal.

8.6 Follow-up of subjects withdrawn from treatment

Normal routinely follow-up at the outpatient clinic, standard control.

8.7 Premature termination of the study

The criteria for terminating the study prematurely are a patient’s wish at any time to discontinue treatment, any unforeseen/unrelated injury that occurs which would require a different treatment. The procedure, basically discontinuing the trial, will depend on the reason of termination. If allowed, we will continue to collect data on these patients according to intention-to-treat principles.

9. SAFETY REPORTING

9.1 Section 10 WMO event

In accordance to section 10, subsection 1, of the WMO, the investigator will inform the subjects and the reviewing accredited METC if anything occurs, on the basis of which it appears that the disadvantages of participation may be significantly greater than was foreseen in the research proposal. The study will be suspended pending further review by the accredited METC, except insofar as suspension would jeopardise the subjects' health. The investigator will take care that all subjects are kept informed.

9.2 Adverse and serious adverse events

Both treatment groups represent variations of standard treatment. We anticipate no treatment related risks related to participation in this study, but it is possible (though unlikely) that one treatment method will prove inferior to the other with respect to functional outcome.

Adverse events are defined as any undesirable experience occurring to a subject during the study. Adverse events are defined as any undesirable experience occurring to a subject during the study. All adverse events, other than pain and joint effusion which are normal findings after arthroscopic knee surgery, by the subject or observed by the investigator or his staff will be recorded.

A serious adverse event is any untoward medical occurrence or effect that at any dose:
- results in death;
- is life threatening (at the time of the event);
- requires hospitalization or prolongation of existing inpatients' hospitalization;
- results in persistent or significant disability or incapacity;
- is a new event of the trial likely to affect the safety of the subjects, such as an unexpected outcome of an adverse reaction, lack of efficacy of an IMP used for the treatment of a life threatening disease, major safety finding from a newly completed animal study, etc.

All SAEs will be reported through the web portal ToetsingOnline to the accredited METC that approved the protocol, according to the requirements of that METC.

SAEs that result in death or are life threatening should be reported expedited. The expedited reporting will occur not later than 7 days after the responsible investigator has first knowledge of the adverse reaction. This is for a preliminary report with another 8 days for completion of the report.

9.3 Suspected unexpected serious adverse reactions (SUSAR)

Not applicable.

9.4 Follow-up of adverse events

All adverse events will be followed until they have abated, or until a stable situation has been reached. Depending on the event, follow up may require additional tests or medical procedures as indicated, and/or referral to the general physician or a medical specialist.

9.5 Data Safety Monitoring Board (DSMB)

Not applicable.

10. STATISTICAL ANALYSIS

Data-analysis and presentation:

To investigate the effect of surgery, we will use generalized estimating equations (GEE) for longitudinal analysis in SPSS 18 on an intention-to-treat and per-protocol basis, as well as sub-group analysis of the cross-over patients. This method takes into account the dependency of observations within a patient, and the fact that not all patients may be assessed at each time point (missing data). In the primary GEE model, the outcome variable studied (e.g. physical function on the IKDC) will be analyzed as a dependent variable, using treatment allocation (1, intervention; 0, control) and time as a key independent variables. In the secondary GEE model, the outcome variables studied (e.g. general health on the RAND36, quality of life on the EQ5D-5L, level of activity on the TAS, knee pain on the question 10 of IKDC, the correlation between a patient's expectation and satisfaction, productivity losses on TiC-P, muscle strength, range of motion, squatting and adverse events) will be analyzed in a similar way. To evaluate whether the two groups differed in change over time the interaction term of group and time (group X time) will be assessed. Time will be included as a dummy variable (reference = baseline T0), and four interaction terms will be analyzed (T1Xgroup; T2x group; T3x group, T4x group). All models will be corrected for center of inclusion and age group. In additional analysis, we will investigate the possible confounding effect (defined as more than 10% change in the parameter estimate for groupXtime) of 5 variables (body mass index, gender, profession, ASA-classification, the affected meniscus, the type of tear and the stadium of OA according to the Kellgren Lawrence Grading Scale for Osteoarthritis.

At the following time points following the treatment (T2, T3, T5 and T7) we will describe the incidence of revisions (intervention group) or treatment failures (=delayed APM, control group) using descriptives. After two years (T7) we will compare the incidence of development or progression of OA between groups using a chi-squared test (or Fisher's Exact as appropriate). For all analysis, a two-tailed value of $p < 0.05$ is considered to be significant.

In all models, missing data (intermittent/drop-out) will be analyzed on being missing completely at random (MCAR) or not (missing at random [MAR]). If not, each model will either be appropriately corrected or missings will be appropriately imputed (to be determined; e.g. last observation carries forward or multiple imputation). In the intention-to-treat analysis we already anticipate a 25% dropout which is non-ignorable (intervention related). Therefore, these data will be analyzed using a weighted GEE analysis. We will consult a statistician for all GEE analysis.

<u>Cost-effectiveness analysis</u>

General considerations:

The economic evaluation will be conducted from a societal perspective. The aim of the economic evaluation is to measure, value and analyze total costs of patients in both groups and to relate the difference in costs between the two treatment groups to the difference in clinical effects. Both a cost-effectiveness and cost-utility analysis will be performed. The time horizon of the economic evaluation is 24 months, so discounting will be used. Sensitivity

analysis will be performed to assess the robustness of the results using different assumptions regarding costs and effects.

Cost-analysis:

Costs will be measured using web-based questionnaires based on the TiC-P at baseline and after 3, 6, 9, 12, 18 and 24 months of follow-up (23). Direct costs include costs of APM surgery and costs of PT, but also other healthcare utilization for knee problems such as GP care, costs of visits to other primary care providers, ambulatory and inpatient hospital care, medication and home care. Indirect costs include absenteeism from paid and unpaid work and presenteeism. The friction cost approach will be used in the primary analysis to estimate indirect costs (27). For the valuation of healthcare utilization standard prices published in the Dutch costing guidelines will be used (28). Medication use will be valued using prices of the Royal Dutch Society for Pharmacy.

Patient outcome analysis:

Effect measures in the economic evaluation are physical function, pain intensity and general health. Quality-adjusted life-years (QALYs) based on the Dutch tariff for the EuroQol will also be measured (22;29).

The analysis will be done according to the intention-to-treat principle. Missing cost and effect data will be imputed using multiple imputation according to the MICE algorithm developed by Van Buuren (30).

Full cost-effectiveness and cost-utility analyzes will be performed. Incremental cost-effectiveness ratios (ICERs) will be calculated by dividing the difference in mean total costs between the treatment groups by the difference in mean effects.

Bias-corrected and accelerated bootstrapping with 5000 replications will be used to estimate 95% confidence intervals around cost differences and the uncertainty surrounding the ICERs. Rubin’s rules will be used to pool the results from the different multiply imputed datasets. Uncertainty surrounding the ICERs will be graphically presented on cost-effectiveness planes.

Cost-effectiveness acceptability curves will also be estimated using the net benefit framework (31). Cost-effectiveness acceptability curves show the probability that APM is cost-effective compared with PT for a range of different ceiling ratios thereby showing decision uncertainty (32).

<u>Budget Impact Analysis</u>

General considerations:

In the budget impact analysis the results of the economic evaluation will be linearly extrapolated over a period of 5 years to estimate the financial consequences of implementation of the study results. An estimate of the long-term financial consequences will also be given to quantify the impact of the expected decrease of the progression of OA and therefore the number of knee arthroplasties. The intervention will be offered to patients aged 45 to 70 years who were diagnosed with symptomatic, non-obstructive, MRI confirmed meniscal tears. Perspectives that will be considered are the societal, government (Budget Kader Zorg) and insurer perspective. Different scenarios will be evaluated including the

following: 1) all patients will receive APM, 2) all patients will receive PT, 3) PT will replace APM gradually over a period of 4 years (25% change per year).
One-way sensitivity analysis will be performed in which the change rate per year and the reduction of number of knee arthroplasties will be varied.

Cost analysis:
The total number of patients aged 45 to 70 years who were diagnosed with symptomatic, non-obstructive, MRI confirmed meniscal tears will be estimated based on Dutch incidence and prevalence rates. Resource utilization is calculated by multiplying the number of eligible patients with the resource utilization rates obtained from the cost-effectiveness analysis.
Different prices will be used to value resource use depending on the perspective of the analysis: Dutch standard costs for the societal perspective, actual NZA tariffs for the government perspective, and average tariffs NZA for the insurer perspective.
Both resource use and annual costs will be presented over a 5 year period for all perspectives. Aggregated and disaggregated total costs per year will be presented for the different perspectives and scenarios. For the long term analysis total costs over the whole time horizon will be estimated.

Data analysts will be blinded to the type of treatment by numerical coding of the performed intervention. After finalizing data analysis this code will be broken for publication purposes.

11. ETHICAL CONSIDERATIONS

11.1 Regulation statement

The study will be conducted according to the principles of the Declaration of Helsinki - 59th WMA General Assembly, Seoul, Korea, October 2008 – and in accordance with the Medical Research Involving Human Subjects Act (WMO).
Because of the study design, that the participators are given the option for delayed surgery if physical therapy fails, no ethical conflicts are expected.

11.2 Recruitment and consent

The treating physician/investigator will approach potential participants about the study during initial visit at the outpatient department. The study will be described in detail and an information letter will be given for patients to read. It will be emphasized that participation is voluntary. Patients are invited, but welcome to decline. Declining to participate in research will not influence their treatment.
Consent will be obtained by the treating physician/investigator during the following visit at the outpatient department, when they return for the result of the MRI. The study protocol will be explained in detail and informed consent form will be signed if patient willing to participate. Subjects will be given a copy of the informed consent form and are informed that they can withdraw at any time during the study.

11.3 Benefits and risks assessment, group relatedness

In this study the current treatment of choice for meniscal injuries, surgery, is compared to physical therapy. In case the conservative approach fails, they will be given the choice to undergo delayed APM. In our view there is no risk for the test person associated with participation in this study. Therefore dispensation for a separate test person insurance for participation in this research is requested with the Medical Ethical Review Committee.
The sponsor/investigator has a liability insurance which is in accordance with article7, subsection 6 of the WMO.

11.4 Compensation for injury

The sponsor/investigator do has a liability insurance which is in accordance with article 7, subsection 6 of the WMO (Article 7 WMO and the Measure regarding Compulsory Insurance for Clinical Research in Humans of 23th June 2003). However, since to our opinion, participation in this trial is without any risks, other than the standard risks associated with the treatment, dispensation from the statutory obligation to provide insurance is requested.

12. ADMINISTRATIVE ASPECTS AND PUBLICATION

12.1 Handling and storage of data and documents

Data will be collected using online questionnaires. All subject data will be anonymised by assigning study numbers to each subject. The study numbers will not be based on the patient initials or birth-date. The key to these study numbers is only available to the coordinating investigator (VG and on demand by the principal investigators). Outcome data, anonymised, is only accessible for the coordinating investigator (VG), principal investigators (RP and AG), statistical analyzers (NW and VS) and authorized research personnel of the JointRearch group at the Onze Lieve Vrouwe Gasthuis Amsterdam. Data will be collected and stored for a period of 15 years.

The original questionnaires will be kept in a database at the initiating hospital (Onze Lieve Vrouwe Gasthuis). Data will be processed and stored in SPSS which will be password protected. Security requirements: Data input capabilities are limited to the coordinating investigator (VG). Data processing capabilities are limited to the coordinating investigator, statistical analyzers (NW and VS), the principal investigators and authorized research staff.

The handling of personal data will comply with the Dutch Personal Data Protection Act (de Wet Bescherming Persoonsgegevens, Wbp).

12.2 Amendments

Amendments are changes made to the research after a favorable opinion by the accredited METC has been given. All amendments will be notified to the METC that gave a favorable opinion. All substantial amendments will be notified to the METC and to the competent authority. Non-substantial amendments will not be notified to the accredited METC and the competent authority, but will be recorded and filed by the sponsor.

12.3 Annual progress report

The sponsor/investigator will submit a summary of the progress of the trial to the accredited METC once a year. Information will be provided on the date of inclusion of the first subject, numbers of subjects included and numbers of subjects that have completed the trial, serious adverse events/ serious adverse reactions, other problems, and amendments.

12.4 End of study report

The investigator will notify the accredited METC of the end of the study within a period of 8 weeks. The end of the study is defined as the last patient’s last visit.

In case the study is ended prematurely, the investigator will notify the accredited METC, including the reasons for the premature termination.

Within one year after the end of the study, the investigator/sponsor will submit a final study report with the results of the study, including any publications/abstracts of the study, to the accredited METC.

12.5 Public disclosure and publication policy

The study will be registered with at least 2 public trial registries ClinicalTrial.gov and BMC Musculoskeletal Disorders.

The outcomes of this study will be used for several purposes:

1) The Royal Dutch Society for Physical Therapy (KNGF; Koninklijk Nederlands Genootschap voor Fysiotherapie), has provided us a statement of adhesion to our project (appendix A). We will discuss our results on regular base with the KNGF.

2) This study will provide best evidence and meaningful information of effectiveness and cost-effectiveness of APM and conservative treatment in terms of PT for symptomatic, non-mechanical meniscal tears in older patients. Policy makers of the Healthcare Insurance Board (College voor Zorgverzekeringen (CVZ)) together will be informed with the necessary data. Depending on the results, a decision could be made for adaptation or consolidation of the current system of reimbursement of this injury.

3) Depending on the results of the proposed study, an implementation plan will be developed. Expertise for the development of such a plan is available among the applicants and the project group has contact with Falke&Verbaan through the consortium of Obstetrics (prof. dr. BW Mol). This independent HRM-organization-advice consultant is specialized in realization of cultural and behavioural change within organizations.

4) The outcome of the proposed study will lead to more specific recommendations in the policy of standard care for meniscal injuries in older patients and will be incorporated in the guidelines of the The Dutch Orthopaedic Association (Nederlandse Orthopaedische Vereniging (NOV)).

5) International publication in peer-reviewed international journal and at congresses.

For authorship regulations, the uniform requirements for manuscripts submitted to biomedical journal as stated by the International Committee of Medical Journal Editors are applied (34). All rights to data and or inventions and Confidential Information shall remain the property of the coordinating investigator at the Onze Lieve Vrouwe Gasthuis (VG). Rights to the data and results, resulting from the performance of the Study (together, “the Study Results”), shall be solely owned by the coordinating investigator at the Onze Lieve Vrouwe Gasthuis (VG).
The Study Results will be published once all subjects have completed the Study and the Study has been analyzed. Publication of results is unrestricted and under the sole authority of the coordinating investigator at the Onze Lieve Vrouwe Gasthuis (VG). The coordinating Investigator (VG) shall be responsible for the general management and supervision of the study.
Authorship credit should be based on 1) substantial contributions to conception and design, or acquisition of data, or analysis and interpretation of data; 2) drafting the article or revising

it critically for important intellectual content; and 3) final approval of the version to be published. Authors should meet conditions 1, 2, and 3. Acquisition of funding, collection of data, or general supervision of the research group, alone, does not justify authorship. All persons designated as authors should qualify for authorship, and all those who qualify should be listed. Each author should have participated sufficiently in the work to take public responsibility for appropriate portions of the content.

13. RISK ANALYSIS

In this trial, we compare a non-invasive intervention, physical therapy, with the current standard treatment, arthroscopic partial meniscectomy.
The specially for this trial designed physical protocol is focused on older patients and is not associated with any risks. The only risks in this study are the risks associated with the current standard treatment, consisting of arthroscopic partial meniscectomy.
Therefore a structured risk analysis had not been performed.

14. REFERENCES

1) van Arkel ERA, van Essen A, et al. Artroscopie van de knie: indicatie en behandeling. Dutch Orthopaedic Association guideline 2011. website Dutch Orthopaedic Association: www.orthopeden.org 2012.
2) Kim S, Bosque J, et al. Increase in outpatient knee arthroscopy in the United States: a comparison of National Surveys of Ambulatory Surgery, 1996 and 2006. J Bone Joint Surg Am 2011 Jun 1;93(11):994-1000.
3) Herrlin SV, Hallander M, et al. Arthroscopic or conservative treatment of degenerative medial meniscal tears: a prospective randomised trial. Knee Surg Sports Traumatol Arthrosc 2007;15(4):393-401.
4) Howell JR, Handoll HH. Surgical treatment for meniscal injuries of the knee in adults. Cochrane Database Syst Rev 2009;(1):CD001353.
5) Mutsaerts EL, van den Bekerom MPJ, et al. Surgical interventions for treating meniscal injuries of the knee in adults (protocol). the Cochrane Collaboration 2010;Issue 6.
6) Herrlin SV, Wange PO, Lapidus G, et al. Is arthroscopic surgery beneficial in treating non-traumatic, degenerative medial meniscal tears. A five year followup. Knee Surg Sports Traumatol Arthrosc 2012;21(2):358-64.
7) Biedert RM, Treatment of intrasubstance meniscal lesions: a randomized prospective study of four different methods. Knee Surg Sports Traumatol Arthrosc 2000; 8(2):104-8.
8) Østeras, H., et al. Medical exercise therapy, and not arthroscopic surgery, resulted in decreased depression and anxiety in patients with degenerative meniscus injury, Journal of Bodywork & Movement Therapies (2012), doi:10.1016/j.jbmt.2012.04.003.
9) Englund M, Guermazi A, et al. Incidental Meniscal Findings on Knee MRI in Middle-Aged and Elderly Persons. New Engl J Med 2008;359:1108-15.
10) Kirkley A, Birmingham TB, et al. Randomized Trial of Arthroscopic Surgery for Osteoarthritis of the Knee. N Engl J Med 2008;359:1097-107.
11) Mosely JB, O'Malley K, et al. A controlled trial of arthroscopic surgery for osteoarthritis of the knee. New Engl J Med 2002;347:81-8.
12) Mesiha M, Zurakowski D, Soriano J, et al. Pathologic characteristics of the torn human meniscus. Am J Sports Med. 2007 Jan;35(1):103-12.
13) Higuchi H, Kimura M, et al. Factors affecting long-term results after arthroscopic partial meniscectomy. Clin Orthop Relat Res 2000 Aug;(377):161-8.
14) Herwig J, Egner E, et al. Chemical changes of human knee joint menisci in various stages of degeneration. Ann Rheum Dis 1984 Aug;43(4):635-40.
15) Kurtz S, Ong K, et al. Projections of primary and revision hip and knee arthroplasty in the United states from 2005 to 2030. J Bone Joint Surg Am 2007;89:780-5.
16) Englund M, Guermazi A, Lohmander LS. The meniscus in knee osteoarthritis. Rheum Dis Clin North Am. 2009 Aug;35(3):579-90.
17) Lexchin J, Bero LA, Djulbegovic B, et al. Pharmaceutical industry sponsorship and research outcome and quality: systematic review. BMJ 2003;326:1167.
18) Crawford K, Briggs KK, Rodkey WG, et al. Reliability, validity, and responsiveness of the IKDC score for meniscus injuries of the knee. Arthroscopy 2007;23(8):839-44.
19) Heijne A, Fleming BC, Renstrom PA, Peura GD, Beynnon BD, Werner S (2004). "Strain on the anterior cruciate ligament during closed kinetic chain exercises." Med Sci Sports Exerc 36(6): 935-41.
20) Heijne A, Werner S. Early versus late start of open kinetic chain quadriceps exercises after ACL reconstruction with patellar tendon or hamstring grafts: a prospective randomized outcome study. Knee Surg Sports Traumatol Arthrosc. 2007;15(4):402-14.
21) Haverkamp D, Sierevelt IN, Breugem SJ, Lohuis K, Blankevoort L, van Dijk CN. Translation and validation of the Dutch version of the International Knee Documentation Committee Subjective Knee Form. Am J Sports Med 2006 Oct;34(10):1680-4.

22) EuroQol Group: EuroQol--a new facility for the measurement of health-related quality of life. The EuroQol Group. Health Policy 1990, 16(3):199-208.
23) Hakkaart-van Roijen L: Handleiding TiC-P (vragenlijst voor zorggebruik en productieverliezen bij psychische aandoeningen). Rotterdam: iMTA, Erasmus Universiteit Rotterdam; 2010.
24) Beurskens AJ, Vet HC de, Köke, Lindeman E, Heijden van der GJ, Regtop W, Knipschild PG. A patient-specific approach for measuring functional status in low back pain. J Manipulative Physiol Ther 1999; 22: 144-8.
25) Tegner Y, Lysholm J. Rating systems in the evaluation of knee ligament injuries. Clin Orthop Relat Res. 1985;198:43-9.
26) Briggs KK, Kocher MS, Rodkey WG, et al. Reliability, Validity, and Responsiveness of the Lysholm Knee Score and Tegner Activity Scale for Patients with Meniscal Injury of the Knee. JBJS Am 2006;88A(4):695-705.
27) Koopmanschap MA, Rutten FF: A practical guide for calculating indirect costs of disease. Pharmacoeconomics 1996,10(5):460-466.
28) Hakkaart-van Roijen L, Tan SS, Bouwmans CAM: Handleiding voor kostenonderzoek: Methoden en standaard kostprijzen voor economische evaluaties in de gezondheidszorg. Geactualiseerde versie 2010. [Dutch manual for costing in economic evaluations]. Diemen: College voor zorgverzekeringen (CVZ); 2011.
29) Lamers LM, Stalmeier PF, McDonnell J, Krabbe PF, van Busschbach JJ: [Measuring the quality of life in economic evaluations: the Dutch EQ-5D tariff]. Ned Tijdschr Geneeskd 2005, 149(28):1574-1578.
30) van Buuren S, Boshuizen HC, Knook DL: Multiple imputation of missing blood pressure covariates in survival analysis. StatMed 1999, 18:681-694.
31) Stinnett AA, Mullahy J: Net health benefits: a new framework for the analysis of uncertainty in cost-effectiveness analysis. Med Decis Making 1998, 18(2 Suppl):68-80.
32) Fenwick E, O'Brien BJ, Briggs A: Cost-effectiveness acceptability curves--facts, fallacies and frequently asked questions. Health Econ 2004, 13(5):405-415.
33) Kellgren JH, Lawrence JS. Radiological assessment of osteo-arthrosis. Ann Rheum Dis. 1957;16(4):494-502.
34) Uniform Requirements for Manuscripts Submitted to Biomedical Journals: Writing and Editing for Biomedical Publication. International Committee of Medical Journal Editors 2008. Ref Type: Internet Communication.
35) American Society of Anesthesiologists. New classification of physical status. Anesthesiology 1963;24:111.

15. APPENDICES

A. Adhesion Royal Dutch Society for Physical Therapy (KNGF)
B. Kellgren Lawrence Grading Scale for Osteoarthritis.
C. Physical therapy protocol
D. ASA physical status classification

Appendix A: Adhesion Royal Dutch Society for Physical Therapy (KNGF)

Postbus 248
3800 AE Amersfoort
Stadsring 159b
3817 BA Amersfoort

T 033 467 29 00
F 033 467 29 99
hoofdkantoor@kngf.nl
www.kngf.nl
www.defysiotherapeut.com

ABN AMRO 55.30.44.028
IBAN NL59ABNA0553044028
BTW 0025.84.700.B.01
KvK 40506528

Retouradres: Postbus 248 - 3800 AE Amersfoort

dr R.W. Poolman (Projectleider en penvoerder)
Onze Lieve Vrouwe Gasthuis
Oosterpark 9
1091 AC Amsterdam

Relatienummer	onbekend	Doorkiesnummer	920
Onze referentie	8072	E-mailadres	vip@kngf.nl
Datum	29 augustus 2012	Pagina	1 van 1
Onderwerp	adhesieverklaring		

Geachte heer van de Graaf,

Met belangstelling heeft het KNGF kennis genomen van uw voornemen om een studie te doen met als titel "Cost-effectiveness of Early Surgery versus Conservative Treatment with Optional Delayed Meniscectomy for Patients over 50 years. A Randomized Controlled Trial.".

Het KNGF verklaart graag adhesie aan uw voornemen en zal met belangstelling kennis nemen van de uitkomsten. Tevens zijn wij waar nodig bereidt een beperkt aantal uren te investeren en de uitkomsten binnen onze beroepsgroep uit te dragen via de gebruikelijke kanalen als FysioEnieuws en Fysiopraxis.

Met vriendelijke groet,

Dr. V.B. de Graaf-Peters (PhD)

Senior Policy Advisor Science, Professional content and Guidelines

de Fysiotherapeut

Koninklijk Nederlands Genootschap voor Fysiotherapie (KNGF)

Stadsring 159b |Postbus 248 | 3800 AE Amersfoort | T 033-4672920 | M 06-22069889|degraafpeters@kngf.nl |www.kngf.nl | www.defysiotherapeut.com

Appendix B: Physical therapy protocol

The exercise program for both groups performed during 8 weeks

Time (week)	Exercises	repetitions or time
**0-8**	stationary bicycling for warming up and cooling down or cardiovasculair training	gradual increase 7-15 min or longer
**0-8**	pully, strap around healty ankle, stay and keep balance on injured side, move healty leg forward, backward and sideward by standing in all 4 directions	3x12
**0-4**	calf raises on a leg press	3x12
**0-8**	standing hip extension in a “multi-hip” trainings device	3x12
**0-4**	balance on wobble board on both feet	
**0-8**	stair walking, walking, running, jumping according the patiënts ICF challanging with throwing a ball	10 min
**5-8**	calf raises standing on one leg	3x12
**1-8**	leg press, place the shinbone horizontal and the knee starting at 110˚, unilateral	3x12
**5-8**	lunges (according the needs of the patiënt) with < 90˚ knee flexion	3x12
**5-8**	balance on wobble board on one foot challing with throwing a ball	3 min
**5-8**	crosstrainer as cardivasculair and cooling down training	10 min or more

footnote:
By all exercises is it important to keep the patiënts individual needs and limitations focused by using the ICF.

The uninjured side is as well less trained as usual and therefor both sides should be trained. Beside the training of the lower extrimity is “core stability” training from importance for good posture positioning and moving.

The active rehabilitation program is designed around cardiovasculaire- (circulation), coordination and balance-, and close-chaine strength exercises. Shearing forces in the knee are less using close-chaine exercises compared to open-chaine exercise. The close-chaine exercise activates both agonisten and antagonisten around the knee joint resulting in a direct rotatory movement and prefends from shearing forces seen by open chaine exercises. (Heijne 2004, 2006 studied the role of open- and close-exercise in the rehabilitation after a reconstruction of the anterior cruciate ligament and advised to be careful with open-chaine exercises in the early start of rehabilitation)

The active rehabilitation program is designed around cardiovasculaire- (circulation), coordination and balance-, and close-chaine strength exercises. Shearing forces in the knee are less using close-chaine exercises compared to open-chaine exercise. The close-chaine exercise activates both agonisten and antagonisten around the knee joint resulting in a direct rotatory movement and prefends from shearing forces seen by open chaine exercises. (Heijne 2004, 2006 studied the role of open- and close-exercise in the rehabilitation after a reconstruction of the anterior cruciate ligament and advised to be careful with open-chaine exercises in the early start of rehabilitation)

In addition, a home program was carried out twice a week in both groups. It consisted of one leg standing during 60 s and a step-down exercise comprising 3 9 10 repetitions

Appendix C: Kellgren Lawrence Grading Scale for Osteoarthritis

Kellgren and Lawrence defined a widely utilized grading system for radiographic evidence of knee OA (28):

- Grade 1: doubtful narrowing of joint space and possible osteophytic lipping
- Grade 2: definite osteophytes, definite narrowing of joint space
- Grade 3: moderate multiple osteophytes, definite narrowing of joint space, some sclerosis and possible deformity of bone contour
- Grade 4: large osteophytes, marked narrowing of joint space, severe sclerosis and definite deformity of bone contour.

Appendix D: ASA physical status classification

American Society of Anesthesiologists' (ASA) physical status classification

Class	Description
I	Healthy patient
II	Mild systemic disease—no functional limitation
III	Severe systemic disease—definite functional limitation
IV	Severe systemic disease that is a constant threat to life
V	Moribund patient unlikely to survive 24 h with or without operation
