# Supplementary material for: Effect of Physical Therapy vs Arthroscopic Partial Meniscectomy in People With Degenerative Meniscal Tears: Five-Year Follow-up of the ESCAPE Randomized Clinical Trial
Source: JAMA Netw Open. 2022 Jul 8;5(7):e2220394. doi: 10.1001/jamanetworkopen.2022.20394 (PMC9270699; doi:10.1001/jamanetworkopen.2022.20394)
Supplement: Supplement 2. — eAppendix. Physical Therapy Protocol eTable. Five-Year Outcomes for Both Treatment Groups [file jamanetwopen-e2220394-s002.pdf]

Supplementary Online Content

Noorduyn JCA, van de Graaf VA, Willigenburg NW, et al; ESCAPE Research Group. Effect of physical therapy vs arthroscopic partial meniscectomy in people with degenerative meniscal tears: five-year follow-up of the ESCAPE randomized clinical trial. *JAMA Netw Open*. 2022;5(7):e2220394. doi:10.1001/jamanetworkopen.2022.20394

eAppendix. Physical Therapy Protocol

eTable. Five-Year Outcomes for Both Treatment Groups

This supplementary material has been provided by the authors to give readers additional information about their work.

© 2022 Noorduyn JCA et al. *JAMA Network Open.*

eAppendix.

Physical therapy protocol

Time (week)	Exercises	repetitions or time
**0-8**	stationary bicycling for warming up and cooling down or cardiovascular training	gradual increase 7-15 min or longer
**0-8**	pully, strap around healthy ankle, stay and keep balance on injured side, move healthy leg forward, backward and sideward by standing in all 4 directions	3x12
**0-4**	calf raises on a leg press	3x12
**0-8**	standing hip extension in a “multi-hip” trainings device	3x12
**0-4**	balance on wobble board on both feet	
**0-8**	stair walking, walking, running, jumping according the patients ICF challenging with throwing a ball	10 min
**5-8**	calf raises standing on one leg	3x12
**1-8**	leg press, place the shinbone horizontal and the knee starting at 110˚, unilateral	3x12
**5-8**	lunges (according the needs of the patient) with < 90˚ knee flexion	3x12
**5-8**	balance on wobble board on one foot challenging with throwing a ball	3 min
**5-8**	crosstrainer as cardiovascular and cooling down training	10 min or more

The exercise program for comprised 16 supervised sessions during 8 weeks

By all exercises is it important to keep the patients individual needs and limitations focused by using the ICF.

The uninjured side is also less trained as usual and therefore both sides should be trained. Besides training of the lower extremity, “core stability” training is of importance for good posture positioning and moving.

The active rehabilitation program is designed around cardiovascular (circulation), coordination and balance, and closed chain strength exercises. Shearing forces in the knee are less using closed chain exercises compared to open chained exercises. The closed chain exercises activate both agonists and antagonists around the knee joint resulting in a direct rotatory movement and prevent in shearing forces seen by open chained exercises. (Heijne 2004, 2006 studied the role of open and closed exercises in the rehabilitation after a reconstruction of the anterior cruciate ligament and advised to be careful with open chained exercises in the early start of rehabilitation).

© 2022 Noorduyn JCA et al. *JAMA Network Open.*

Home exercise program

In addition, a home exercise program was provided to all participants. It consisted of one leg standing during 60 seconds and a step-down exercise comprising 3, 9, 10 repetitions, twice a week.

© 2022 Noorduyn JCA et al. *JAMA Network Open.*

eTable. Five-year outcomes for both treatment groups.

	Meniscal surgery group	Physical therapy group
Patient reported outcome	n= 139	n= 139
Women	73 (52.5%)	71 (51.1%)
IKDC score	74.7 ±18.4	73.1 ±17.7
Pain during activities	19.0 ±25.0	20.0 ±24.0
KOOS-PS	19.9 ±16.8	22.5 ±15.0
EQ-5D-5L	0.87 ±0.15	0.87±0.14
Knee OA on radiographs	n=112	n=110
OARSI sum score	3.0 ±2.6	3.4 ±2.7
Progression on OARSI *Baseline – 5 years*	1.1 ±2.2	1.1 ±2.1
KL classification[c]		
0 - No OA	8 (7.1%)	8 (7.3%)
1 - Doubtful OA	44 (39.3%)	39 (35.5%)
2 - Minimal OA	31 (27.7%)	33 (30.0%)
3 - Moderate OA	18 (16.1%)	18 (16.4%)
4 - Severe OA[d]	11 (9.8%)	12 (10.9%)
Symptomatic knee OA *yes (%)*	4 (3.6%)	2 (1.9%)
Additional knee surgeries[e]		
Delayed surgery	NA	52 (37.4%)
Meniscal re-surgery	2 (1.4%)	6 (4.3%)
TKP or UKP	3 (2.2%)	4 (2.9%)
Cartilage surgery	0 (0%)	1 (0.7%)
Arthroscopic inspection	0 (0%)	1 (0.7%)

Data are n (%) or mean ± SD.

Abbreviations: IKDC=International Knee Documentation Committee, KOOS-PS = Knee Osteoarthritis Outcome Score – physical function short form, EQ-5D-5L = EuroQol 5 Dimension 5 Level, OA=Osteoarthritis, OARSI = Osteoarthritis Research Society International, KL=Kellgren-Lawrence classification, TKR = total knee replacement, UKP = Unicompartal knee prosthesis.

[e] In the PT group 12 patients underwent additional knee surgery, other than delayed surgery. However, of these 12 patients, 10 patients first had a delayed surgery.

© 2022 Noorduyn JCA et al. *JAMA Network Open.*
