# Supplementary material for: Effect of Physical Therapy vs Arthroscopic Partial Meniscectomy in People With Degenerative Meniscal Tears: Five-Year Follow-up of the ESCAPE Randomized Clinical Trial
Source: JAMA Netw Open. 2022 Jul 8;5(7):e2220394. doi: 10.1001/jamanetworkopen.2022.20394 (PMC9270699; doi:10.1001/jamanetworkopen.2022.20394)
Supplement: Supplement 3. — Data Sharing Statement [file jamanetwopen-e2220394-s00.pdf]

Data Sharing Statement

Noorduyn. Effect of Physical Therapy vs Arthroscopic Partial Meniscectomy in People With Degenerative Meniscal Tears. *JAMA Netw Open*. Published July 08, 2022. doi:10.1001/jamanetworkopen.2022.20394

Data
Data available: Yes
Data types: Deidentified participant data
How to access data: Requests for access to data should be addressed to the corresponding author (j.c.a.noorduyn@olvg.nl).
When available: With publication

Supporting Documents
Document types: None

Additional Information
Who can access the data: researchers whose proposed use of the data has been approved
Types of analyses: Meta analyses, prediction models
Mechanisms of data availability: after approval of a proposal and with a signed data access agreement
